# Multi-omics analysis uncovered systemic lupus erythematosus and COVID-19 crosstalk

**DOI:** 10.1186/s10020-024-00851-6

**Published:** 2024-06-11

**Authors:** Zekai Nian, Yicheng Mao, Zexia Xu, Ming Deng, Yixi Xu, Hanlu Xu, Ruoyao Chen, Yiliu Xu, Nan Huang, Feiyang Mao, Chenyu Xu, Yulin Wang, Mengyuan Niu, Aqiong Chen, Xiangyang Xue, Huidi Zhang, Gangqiang Guo

**Affiliations:** 1 Second Clinical College, Wenzhou Medical University, Wenzhou, China; 2 Ophthalmology College, Wenzhou Medical University, Wenzhou, China; 3 Department of Nephrology, First Affiliated Hospital, Wenzhou Medical University, Wenzhou, China; 4 Public Health and Management College, Wenzhou Medical University, Wenzhou, China; 5 School of Public Administration, Hangzhou Normal University, Hangzhou, China; 6 Research Center of Fluid Machinery Engineering and Technology, Jiangsu University, Zhenjiang, China; 7 Wenzhou Collaborative Innovation Center of Gastrointestinal Cancer in Basic Research and Precision Medicine, Wenzhou Key Laboratory of Cancer-Related Pathogens and Immunity, Department of Microbiology and Immunology, Institute of Molecular Virology and Immunology, Institute of Tropical Medicine, School of Basic Medical Sciences, Wenzhou Medical University, Wenzhou, China; 8 Department of Rheumatology, Ningbo Medical Center Lihuili Hospital, Ningbo, China

**Keywords:** COVID-19, Cytokine release syndrome, Monokine, Interferon, JAK-STAT, Systemic lupus erythematosus

## Abstract

**Background:**

Studies have highlighted a possible crosstalk between the pathogeneses of COVID-19 and systemic lupus erythematosus (SLE); however, the interactive mechanisms remain unclear. We aimed to elucidate the impact of COVID-19 on SLE using clinical information and the underlying mechanisms of both diseases.

**Methods:**

RNA-seq datasets were used to identify shared hub gene signatures between COVID-19 and SLE, while genome-wide association study datasets were used to delineate the interaction mechanisms of the key signaling pathways. Finally, single-cell RNA-seq datasets were used to determine the primary target cells expressing the shared hub genes and key signaling pathways.

**Results:**

COVID-19 may affect patients with SLE through hematologic involvement and exacerbated inflammatory responses. We identified 14 shared hub genes between COVID-19 and SLE that were significantly associated with interferon (IFN)-I/II. We also screened and obtained four core transcription factors related to these hub genes, confirming the regulatory role of the IFN-I/II-mediated Janus kinase/signal transducers and activators of transcription (JAK-STAT) signaling pathway on these hub genes. Further, SLE and COVID-19 can interact via IFN-I/II and IFN-I/II receptors, promoting the levels of monokines, including interleukin (IL)-6/10, tumor necrosis factor-α, and IFN-γ, and elevating the incidence rate and risk of cytokine release syndrome. Therefore, in SLE and COVID-19, both hub genes and core TFs are enriched within monocytes/macrophages.

**Conclusions:**

The interaction between SLE and COVID-19 promotes the activation of the IFN-I/II-triggered JAK-STAT signaling pathway in monocytes/macrophages. These findings provide a new direction and rationale for diagnosing and treating patients with SLE–COVID-19 comorbidity.

**Supplementary Information:**

The online version contains supplementary material available at 10.1186/s10020-024-00851-6.

## Background

Coronavirus disease (COVID-19), a pneumonia caused by severe acute respiratory syndrome coronavirus 2 (SARS-CoV-2), has resulted in nearly 7 million deaths (Number of COVID-19 deaths reported to WHO (cumulative total) 2024). While knowledge about the intricate pathogenesis of COVID-19 is limited, increasing evidence suggests that the dysregulation of immune responses and excessive production of cytokines following SARS-CoV-2 infection are key reasons for COVID-19 onset and secondary multi-organ damage (Lowery et al. 2021). Furthermore, various autoantibodies, such as antiphospholipid and antinuclear antibodies, have been identified in patients with COVID-19 (Liu et al. 2021). These findings underscore notable similarities between the pathogenic mechanisms of COVID-19 and autoimmune diseases (ADs). Patients with COVID-19 subsequently develop autoimmune disorders, such as immune thrombocytopenic purpura, autoimmune hepatitis, and systemic lupus erythematosus (SLE) (Liu et al. 2021; Garrido et al. 2021; Bonometti et al. 2020); thus, in addition to posing a severe burden on global public health systems, COVID-19 presents a significant challenge in AD management (Ferri et al. 2020). As such, many rheumatologists are now speculating whether patients with ADs that contract COVID-19 experience an exacerbation of the autoimmune disorders and more severe adverse outcomes related to COVID-19 (Liu et al. 2021).

SLE, one of the most common ADs, is characterized by multi-systemic and multi-organ involvement, recurrent relapses and remissions, and the presence of numerous autoantibodies in the body (Crow 2023). The etiology and pathogenesis of SLE are associated with various factors, including infections, genetics, and hormonal abnormalities. Among these, viral infections such as those caused by Epstein-Barr virus, cytomegalovirus, and mumps virus are crucial in SLE onset and progression (Kaul et al. 2016). Studies have highlighted the close connection between the pathogenesis of COVID-19 and SLE. For instance, both COVID-19 and SLE present with increased numbers of immune cells such as monocytes, macrophages, mast cells, and neutrophils and elevated levels of cytokines, including interleukin (IL)-6, IL-10, tumor necrosis factor-α (TNF-α), interferon-α (IFN-α), IFN-β, and IFN-γ (Liu et al. 2021). Furthermore, a study has reported that the incidence rates (IR) for COVID-19-associated hospitalization were higher in patients with SLE [IR ratio (IRR) 2.0 (1.4–2.7)) compared with the general population (Bournia et al. 2023). Meanwhile, COVID-19-associated organ failure has also been found to be associated with a poor late-onset prognosis among patients with SLE (Mageau et al. 2022). Thus, understanding how COVID-19 affects the progression of SLE and identifying potential therapeutic approaches targeting the interaction between SLE and COVID-19 are essential. However, few studies have reported the potential interactive mechanisms between SLE and COVID-19.

Herein, clinical information of patients with SLE–COVID-19 comorbidity and related ribonucleic acid-sequencing (RNA-seq) datasets, genome-wide association study (GWAS) datasets, and single-cell RNA (scRNA)-seq datasets were used to elucidate the potential interplay mechanisms between both diseases.

## Methods

### Patient selection

We retrospectively collected clinical information of 50 patients with SLE (49 female) and 110 SLE patients (101 female) infected with SARS-CoV-2 (SARS-CoV-2 infection confirmed by positive testing through SARS-CoV-2 nucleic acid swabs) treated in the outpatient department of the First Affiliated Hospital of Wenzhou Medical University (hereafter referred to as our hospital) from December 2022 to March 2023. We also collected clinical data from 26 hospitalized patients with COVID-19 (patients with SARS-CoV-2 nucleic acid swabs positive and CT images showing viral pneumonia) and 34 patients with SLE (30 female) treated in our hospital before and after contracting COVID-19. The collected data included patients’ demographic information and clinical and laboratory examination results, including clinical manifestations, comorbidities, medication administration, and serological test results such as cytokine levels. Laboratory test results were collected during the last follow-up visit before the contraction of COVID-19 and the first follow-up visit after COVID-19 infection, wherever possible. In this study, combined with the newest guidelines, we classified comorbidity patients as having mild or moderate/severe cases based on whether they required at least double the dose of glucocorticoids to meet treatment needs, and/or the SLE Disease Activity Index 2000 (SLE-DAI-2 K) score (> 4) (Fanouriakis et al. 2021; Fanouriakis et al. 2024). Furthermore, based on a previous study, we diagnosed patients with COVID-19 and SLE–COVID-19 with cytokine release syndrome (CRS) if they met one or more of the following diagnostic criteria: (1) C-reactive protein (CRP) > 100 mg/L, (2) lymphocyte count < 0.6 × 109/L, (3) serum IL-6 levels ≥ 18 pg/mL, (4) ferritin > 600 μg/L and lactate dehydrogenase > 250 U/L, and (5) D-dimer > 1 μg/mL (Memish et al. 2021).

### RNA-seq datasets

All RNA-seq datasets included in this study were obtained from the GEO database (https://www.ncbi.nlm.nih.gov/geo/) (Barrett et al. 2011) (Additional file 1: Table S1). The datasets mainly comprise the following: (1) 212 COVID-19 and 392 SLE patients: Bulk RNA-seq datasets including four sets of SLE patient data (GSE50772, GSE121239, GSE122459, and GSE139940) and three sets of COVID-19 patient data (GSE179850, GSE164805, and GSE186460). Specifically, the GSE50772, GSE121239, and GSE179850 datasets were used for weighted correlation network analysis (WGCNA) and as the training sets for building machine learning (ML) models. The GSE122459 and GSE164805 datasets served as the external validation sets for the ML models. GSE186460 and GSE139940 were used to validate the activation of the Janus kinase/signal transducers and activators of the transcription (JAK-STAT) pathway in patients with COVID-19 and SLE. Additionally, part of the GSE161664 dataset was divided into six groups: a healthy control (HC) group comprising four healthy samples; IFN-α, IFN-β, and IFN-γ groups, each consisting of three samples treated with IFN-α, IFN-β, or IFN-γ, respectively; and groups treated with IFN-β + baricitinib or IFN-β + ruxolitinib, each containing three samples. In addition, scRNA-seq datasets included the SLE-related dataset GSE162577 and the COVID-19-related dataset GSE158055; and (2) 338 Pan-AD patients: Thirteen bulk RNA-seq datasets (including 16 prevalent ADs), including GSE25101 (ankylosing spondylitis), GSE3365 (Crohn’s disease and ulcerative colitis), GSE113469 (celiac), GSE112943 (cutaneous lupus erythematosus and lupus nephritis), GSE128470 (dermatomyositis), GSE159225 (multiple sclerosis), GSE21592 (narcolepsy), GSE128470 (necrotizing myopathy and polymyositis), GSE192867 (psoriasis), GSE90081 (rheumatoid arthritis), GSE40611 (Sjögren’s syndrome), GSE117928 (systemic sclerosis), and GSE156035 (type 1 diabetes).

### GWAS datasets

Fifty-two GWAS datasets (4,698,024 COVID-19 and 879,441 SLE patients) were obtained from the integrative epidemiology unit (IEU) OpenGWAS project (https://gwas.mrcieu.ac.uk/) database (Additional file 1: Table S2). Single nucleotide polymorphisms (SNPs) with a genome-wide significance (P = 5 × 10^−8^, r2 = 0.001, kb = 10,000) were used as instrumental variables. We incorporated all results obtained from the IEU database with the keywords ‘SLE,’ ‘COVID-19,’ ‘IFN-I/II,’ ‘JAK1/2,’ ‘TYK2,’ ‘STAT1/2,’ and ‘IRF7/9’ and their synonymous expressions; filtered out datasets that fit our research interest; and integrated the results of data set collection into two groups: one for molecules (JAKs, STATs, IFN regulatory factors (IRFs), and IFNs) and another for diseases (SLE and COVID-19).

### Processing of bulk RNA-seq datasets

After downloading related bulk RNA-seq datasets from the GEO database, we performed log2 processing and probe annotation on the raw data using the Pandas library in Python. For research purposes, we merged GSE50772 and GSE122459 into a dataset to obtain a large sample size and used the Combat function from the sva package in R (V4.1.3) to eliminate batch differences.

### WGCNA

WGCNA is an algorithm that constructs co-expression gene modules through the co-expression associations among genes and analyzes the relevance between a gene module and its biological phenotypes (Langfelder and Horvath 2008). The ImageGP (http://www.ehbio.com/Cloud_Platform/front/#/) website (Chen et al. 2022) was used for online WGCNA, under the following parameters: Correlation algorithms = Pearson, Network type = Unsigned, Remove outlier samples = TRUE (remove), Outlier sample detection threshold = -2.5, R square cut = 0.85, Specific maximum power to check = 20, selection of only top genes with maximum mean absolute deviations (MAD) = 2000, Minimum module size for module detection = 20, Deep split = 2.

### Construction of protein–protein interaction (PPI) network and module analysis

The Search Tool for the Retrieval of Interacting Genes (STRING) database (http://string-db.org) (Szklarczyk et al. 2019) was used to construct the PPI network. Using the Molecular Complex Detection (MCODE) plugin (Bader and Hogue 2003) in the Cytoscape software (Shannon et al. 2003), subnetworks were selected based on the information on edges and nodes in the interaction network. Parameter settings for MCODE were as follows: degree cut = 2, max depth = 100, node score cut = 0.2, K-core = 2. GeneMANIA (http://genemania.org) (Franz et al. 2018) was used to predict gene functions and gene interactions, with the following parameters: Max resultant genes = 20, Max resultant attributes = 10, Network weighting = Automatically selected weighting method.

### Gene Ontology (GO) and pathway enrichment analysis

Database for Annotation, Visualization, and Integrated Discovery (DAVID, http://david.abcc.ncifcrf.gov/home.jsp) (Huang et al. 2009) was used for GO enrichment and pathway analyses. The pathway analysis results were obtained from the Kyoto Encyclopedia of Genes and Genomes (KEGG), WIKI, and REACTOME databases. Results were considered significant at a P-value < 0.05.

### Evaluation of applicant drugs

Target-specific drug molecules that could inhibit the expression of target genes were identified through the online website Enrichr (Kuleshov et al. 2016) and the Connectivity Map (CMap) database. The reliability of these drug molecules was also further evaluated using previously described methods (Yoo et al. 2015).

### Construction of ML model

All ML programs were run in Python. We divided the datasets used for ML application into training and validation sets at a 7:3 ratio. For each training and validation set, we applied eight ML algorithms—Logistic Regression, Decision Tree, Random Forest, Neural Network, Bayes, Support Vector Machine, Extreme Gradient Boosting, and K-Neighbor, all using default parameters. Extreme Gradient Boosting was performed using the XGBoost package, while the others were performed using scikit-learn. The model_selection package from scikit-learn was used to divide the data into five different training sets and test sets, and five-fold cross-validation was performed for each model. Receiver operating characteristic (ROC) and macro-average ROC curves of each fold in the cross-validation were plotted.

### Identification of transcription factors (TFs)

ChEA3 (https://amp.pharm.mssm.edu/ChEA3) (Keenan et al. 2019) was used to identify TFs related to hub genes. The top-ranked entries were selected from the integrated results of all libraries in ChEA3 to identify TFs most correlated with the input genes.

### Two-sample Mendelian randomization (MR) analysis

All MR analyses were conducted in R (V4.1.3). Except for the ld_clump_local function, which is from the ieugwasr package, all other functions described below were from the TwoSampleMR package. In both two-sample MR analyses, either SLE- or COVID-19-related datasets were set as exposure or outcomes. Meanwhile, molecular or disease datasets were used as another set of exposure or outcomes. For exposure data, the parameter was set as P1 = 5e−08 to ensure a strong correlation of SNPs with the exposure factor. Limitations for parameters r2 = 0.001 and kb = 10,000 were also applied to control for the linkage disequilibrium (LD) among variables. The exposure data was clumped using the ld_clump_local function and the plinkbinr package based on the bfile of 1000 genomes reference panels for LD for each superpopulation. After obtaining the outcome data and formatting, the Wald ratio method was used to compute the MR estimates for each SNP. When more than one SNP was available, a weighted mean of the ratio estimates, weighted by the inverse variance weighted (IVW), was used (Su et al. 2023). MR analysis was performed using IVW, MR–Egger, weighted median, simple mode, and weighted mode methods, while sensitivity analyses were conducted using leave-one-out analysis. We assessed whether the MR–Egger intercept significantly deviated from 0 to test horizontal pleiotropy when the number of SNPs was no less than 3. Cochran’s Q test was used to test for heterogeneity between Wald ratios.

### Process and analysis of scRNA-seq datasets

The processing and analysis of scRNA-seq datasets related to SLE were mainly completed using the Seurat package in R (V4.1.3). First, the data was normalized. Then, quality control was performed based on the criteria that the number of RNA species should be greater than 500 and less than 3,000, and the proportion of mitochondrial genes should not exceed 10% (Additional file 1: Figure S1A). Next, linear normalization and principal component analysis (PCA) were conducted, and the first 15 principal components were selected for subsequent analyses (Additional file 1: Figure S1B). Cells were clustered using the FindClusters function, and dimensionality reduction of the principal components was achieved through the t-distributed stochastic neighbor embedding (TSNE) algorithm. Finally, cell annotations were performed based on the HumanPrimaryCellAtlasData reference dataset in the SingleR package, and scatter plots with cell annotations were plotted using the DimPlot function. Plots depicting the expression levels of respective genes were created using the FeaturePlot function. All analyses and visualization of the COVID-19 scRNA-seq data were achieved using the GSE158055 dataset and its online website (http://covid19.cancer-pku.cn/#/summary).

### Statistical analysis

All statistical analyses were performed using SPSS (Statistical Product and Service Solutions) software. Changes in the expression levels of hub genes in SLE and COVID-19 and the regulatory role of the JAK-STAT pathway on these hub genes were validated using the unpaired t-test. The clinical information was descriptively analyzed using medians, quartiles, and percentages. Additionally, a paired-sample t-test was performed on data from patients with SLE before and after contracting COVID-19. Other clinical information was analyzed using the chi-squared test and the non-parametric Mann–Whitney test for between-group comparisons.

## Results

### Clinical features of the crosstalk between COVID-19 and SLE highlight the immune disorder

To comprehensively elucidate the impact of COVID-19 on SLE, we performed four comparative analyses examining the changes in clinical information of SLE outpatients with or without SARS-CoV-2 infection, SLE outpatients with SARS-CoV-2 infection of varying severity, hospitalized SLE patients before and after contracting COVID-19, and hospitalized patients with SLE–COVID-19 of varying severity. First, compared with patients with SLE, SLE outpatients with SARS-CoV-2 infection had decreased platelet count (PLC), white blood cell (WBC) count, urinary leukocyte count, and use of immunosuppressants (Additional file 1: Table S3). Furthermore, hospitalized patients with SLE post-COVID-19 exhibited reduced eosinophil, basophil, NK cell, and CD3^+^, CD4^+^, and CD8^+^ T cell counts and high-density lipoprotein levels, while the levels of C-reactive protein (CRP), N-terminal pro-B-type natriuretic peptide (NT-prBNP), complement (C) 4 levels and CD3-CD19^+^ B cell count increased. Additionally, the use of immunosuppressants decreased while that of antivirals, antibiotics, and traditional Chinese medicine preparations increased (Table 1 and Additional file 1: Table S4). The C3 level was relatively lower in outpatients with moderate/severe SLE–SARS-CoV-2 than in patients with mild SLE–SARS-CoV-2, while the percentage of anti-dsDNA positivity and use of glucocorticoids was higher (Additional file 1: Table S5). In hospitalized SLE–COVID-19 patients with moderate/severe disease, the PLC, albumin, and C3 levels, and CD3^+^ T cell count were reduced, while the disease activity index 2000 (SLE-DAI-2 K) score and use of immunosuppressants was elevated (Table 2). These findings suggest that the impact of SARS-CoV-2 and COVID-19 on SLE patients is similar, and that COVID-19 has a more significant impact on SLE patients than SARS-CoV-2 infection by exacerbating the inflammatory response in SLE patients and increasing the risk of secondary organ damage, especially cardiac injury. In addition, hematologic involvement was significant in both diseases. However, the high disease activity level in SLE patients is also associated with the deterioration of comorbid cases.

**Table 1:** Clinical information comparison between hospitalized patients with SLE before and after contracting COVID-19 (unpaired samples)

Patients	SLE, N = 28		SLE + COVID-19, N = 34^#^		P-value
*SLE-DAI-2 K score (median, 25%-75%)*	3 (2–6)			2 (1–4)	0.223
*Laboratory examination (median, 25%-75%)*					
*Blood routine examination*					
RBC	3.84 (3.03–4.35)		3.50 (2.99–4.09)		0.432
HGB	117 (84–127)		105 (88–120)		0.343
PLC	173 (103–214)		146 (113–199)		0.646
WBC	5.81 (4.55–7.56)		4.75 (3.21–7.67)		0.161
AEC	0.020 (0.010–0.100)		0.000 (0.000–0.010)		< 0.001***
ANC	3.19 (2.02–6.11)		3.65 (2.11–5.79)		0.938
AMC	0.64 (0.38–0.91)		0.31 (0.21–0.52)		0.273
ABC	0.010 (0.003–0.020)		0.000 (0.000–0.010)		0.010*
CRP, N = 31/16^†^	2.7 (1.45–12.0)		25.2 (16.5–76.1)		0.002**
ESR, N = 17/24^†^	16.0 (4.0–24.5)		18.5 (11.0–32.3)		0.177
*Biochemical examination of blood component*					
Total bilirubin, N = 23/30^†^	9 (7–12)		8 (6–11)		0.458
Albumin, N = 25/31^†^	35.1 (29.9–38.6)		31.1 (26.9–36.6)		0.053
ALT, N = 27/34^†^	16 (12–28)		18 (11–30)		0.668
AST, N = 27/33^†^	21(17–26)		26 (20–39)		0.022*
ALP, N = 24/31^†^	62 (45–87)		63 (48–88)		0.605
GGT, N = 24/31^†^	25 (17–57)		32 (22–73)		0.172
Urea, N = 27/34^†^	9.3 (5.1–15.6)		8.5 (4.5–14.3)		0.566
Creatinine, N = 27/34^†^	105 (61–207)		89 (57–272)		0.766
Uric acid, N = 26/28^†^	327 (241–462)		318 (260–423)		0.931
Serum potassium, N = 25/33^†^	4.11 (3.61–4.50)		3.72 (3.47–4.27)		0.158
Serum sodium, N = 25/33^†^	139 (136–141)		139 (136–142)		0.981
Serum calcium, N = 20/28^†^	2.19 (2.07–2.32)		2.13 (2.04–2.23)		0.202
Serum phosphate, N = 17/19^†^	1.36 (1.10–1.66)		1.07 (0.90–1.38)		0.079
Total cholesterol, N = 21/25^†^	4.86 (4.24–5.71)		4.45 (3.55–5.26)		0.081
Triglycerides, N = 21/25^†^	1.70 (1.00–2.50)		1.80 (1.06–2.55)		0.732
HDL-C, N = 21/25^†^	1.32 (1.05–1.58)		1.01 (0.93–1.22)		0.004**
LDL-C, N = 21/25^†^	2.66 (2.34–3.18)		2.57 (1.98–3.43)		0.537
Urinary protein, N = 20/28†	9 (45.0)		9 (32.1)		0.364
LEU, N = 25/27^†^	6 (3–25)		7 (2–46)		0.769
BNP, N = 0/10^†^	-		63.0 (27.5–345.0)		-
NT-prBNP, N = 21/5^†^	113.0 (43.3–204.5)		429.0 (108.1–4833.5)		0.034*
*Immune-related*					
IgA, N = 27/25^†^	1.77 (1.31–2.85)		1.99 (1.47–2.38)		0.647
IgG, N = 27/25^†^	10.30 (8.62–13.64)		9.91 (8.19–12.46)		0.510
IgM, N = 27/25^†^	0.66 (0.48–1.19)		0.73 (0.44–1.47)		0.977
C3, N = 26/25^†^	0.78 (0.60–1.00)		0.91 (0.72–1.21)		0.141
C4, N = 22/24^†^	0.22 (0.10–0.27)		0.30 (0.19–0.43)		0.049*
Complement deficiency, N = 26/25^†^	9 (34.6)		6 (24.0)		0.406
Anti-dsDNA antibodies, N = 18/24^†^	5 (27.8)		7 (29.2)		0.921
*Cell composition, N = 11/22*^†^					
CD3 + T cells (%)	84.1 (75.3–89.1)		71.9 (59.7–85.6)		0.048*
CD4 + T cells (%)	35.9 (26.8–51.9)		30.6 (26.3–36.4)		0.299
CD8 + T cells (%)	48.9 (37.2–54.4)		41.5 (29.5–56.7)		0.585
CD4 + T cells/CD8 + T cells	0.71 (0.55–1.40)		0.75 (0.59–1.13)		0.985
CD3-CD19 + B cells (%)	5.4 (3.5–7.1)		8.4 (2.8–17.0)		0.063
CD3-CD56 + CD16 + NK cells (%)	11.4 (4.2–18.2)		14.4 (7.2–22.4)		0.355
T lymphocyte, N = 10/19^†^	496 (366–1135)		228 (184–616)		0.045*
CD4 + T lymphocyte, N = 10/19^†^	188 (136–673)		136 (63–184)		0.040*
CD8 + T lymphocyte, N = 10/19^†^	287 (227–576)		125 (89–348)		0.033*
B lymphocyte, N = 10/19^†^	34 (11–76)		23 (11–76)		0.804
NK cell, N = 10/19^†^	58 (39–239)		53 (27–74)		0.330
*Medication administration, N (%)*					
Glucocorticoids	28 (100.0)		34 (100.0)		-
Hydroxychloroquine	16 (57.1)		17 (50.0)		0.575
Immunosuppressants	16 (57.1)		11 (32.4)		0.049*
Antiviral	2 (7.1)		23 (67.6)		< 0.001***
Antibiotics	8 (28.6)		23 (67.6)		0.002**
Antipyretic analgesics	0		6 (17.6)		0.022*

*ALT* alanine transaminase, *AST* aspartate transaminase, *ALP* alkaline phosphatase, *GGT* gamma-glutamyl transferase, *HDL-C* high-density lipoprotein cholesterol, *LDL-C* low density lipoprotein cholesterol, *LEU* urinary leukocyte count, *BNP* B-type natriuretic peptide, *NT-prBNP* N-terminal pro-B-type natriuretic peptide, *IgA* immunoglobulin A, *IgG* immunoglobulin G, *IgM* immunoglobulin M, *C3* complement 3, *C4* complement 4, *NK* natural killer, *RBC* red blood cell, *HGB* hemoglobin, *PLC* platelet count, WBC white blood cell, *AEC* absolute eosinophil count, *ANC* absolute neutrophil count, *AMC* absolute monocyte count, *ABC* absolute basophil count, *CRP* C-reactive protein, *ESR* erythrocyte sedimentation rate

^#^6 SLE patients lacked clinical information before merging with COVID-19 from their first visit to our hospital

^†^N = a/b indicates that this examination came from a) relieved SLE–COVID-19 comorbidity patients and b) worsened comorbidity patients

^*^, P < 0.05; ^**^, P < 0.01; ^***^, P < 0.001

**Table 2:** Clinical information comparison between hospitalized patients with mild or moderate/severe SLE–COVID-19

SLE–COVID-19 comorbidity patients	Mild (N = 29)	Moderate/severe (N = 5)	P-value
*Demographics*			
Age, y, mean (25–75%)	55.5 (44–59)	43 (27–53)	0.293
Gender, female, N (%)	25 (86.2)	5 (100.0)	0.512
*Clinical manifestations of COVID-19, N (%)*			
Fever	21 (72.4)	5 (100.0)	0.236
Cough and/or expectoration	26 (89.7)	3 (60.0)	0.146
Lassitude	8 (27.6)	2 (40.0)	0.465
Muscle soreness	5 (17.2)	1 (20.0)	0.647
Runny and stuffy nose	3 (10.3)	0	0.611
Pharyngalgia and itchy throat	4 (13.8)	0	0.512
Abdominal pain and diarrhea	2 (6.9)	0	0.724
Nausea and vomiting	4 (13.8)	0	0.512
Tachypnea and chest distress	10 (34.5)	1 (20.0)	0.471
Dizziness and headache	1 (3.4)	0	0.853
*SLE-DAI-2 K score (median, 25–75%)*	1 (1–2.5)	11 (8.5–13.5)	< 0.001***
*Comorbidities, N (%)*			
Hypertension	20 (69.0)	2 (40.0)	0.225
Diabetes	5 (17.2)	0	0.427
PAH	3 (10.3)	1 (20.0)	0.488
Cardio-cerebrovascular disease	7 (24.1)	0	0.290
*Laboratory examination (median, 25–75%)*			
*Blood routine examination*			
RBC	3.55 (3.06–4.27)	2.25 (1.79–3.60)	0.710
HGB	107 (94–121)	70 (53–106)	0.050
PLC	160 (130–208)	62 (20–95)	0.001**
WBC, N = 28/5^†^	4.75 (3.27–7.92)	4.09 (1.89–5.63)	0.419
AEC	0.000 (0.000–0.010)	0.000 (0.000–0.020)	0.741
ANC	3.67 (2.26–6.18)	2.84 (0.99–4.33)	0.135
AMC	0.31 (0.20–0.51)	0.26 (0.11–0.62)	0.637
ABC	0.005 (0.000–0.010)	0.000 (0.000–0.005)	0.232
CRP, N = 27/4^†^	28.3 (17.1–76.1)	15.0 (6.5–96.8)	0.408
ESR, N = 21/3^†^	17.0 (10.5–31.5)	29.0 (21.0–31.0)	0.505
*Biochemical examination of blood component, N = 26/5*			
Total bilirubin, N = 26/5^†^	8 (6–12)	8 (7–10)	0.957
Albumin, N = 26/5^†^	32.8 (28.6–37.8)	24.7 (19.4–27.9)	0.004**
ALT	17 (11–30)	19 (11–114)	0.603
AST, N = 28/5^†^	26 (20–34)	27 (20–181)	0.419
ALP, N = 26/5^†^	63 (51–86)	82 (35–166)	0.696
GGT, N = 26/5^†^	31 (22–58)	117 (17–448)	0.358
Urea	7.7 (4.4–14.4)	9.7 (5.7–15.7)	0.706
Creatinine	83 (60–283)	116 (51–157)	0.671
Uric acid, N = 24/4^†^	293 (249–423)	375 (281–431)	0.547
Serum potassium, N = 28/5^†^	3.76 (3.46–4.32)	3.69 (3.34–4.40)	0.942
Serum sodium, N = 28/5^†^	138 (135–142)	140 (133–142)	0.827
Serum calcium, N = 25/3^†^	2.13 (2.04–2.23)	2.07 (1.96–2.10)	0.280
Serum phosphate, N = 17/2^†^	1.07 (0.83–1.74)	1.01 (0.91–1.11)	0.842
Total cholesterol, N = 22/3^†^	4.47 (3.60–5.44)	3.69 (3.02–4.17)	0.398
Triglycerides, N = 22/3^†^	1.73 (1.06–2.51)	1.93 (1.04–2.66)	0.906
HDL-C, N = 22/3^†^	1.02 (0.94–1.28)	1.00 (0.75–1.08)	0.663
LDL-C, N = 22/3^†^	2.60 (1.98–3.55)	2.33(1.75–2.83)	0.606
Urinary protein, N = 23/5^†^	6 (26.1)	3 (60.0)	0.290
LEU, N = 23/4^†^	5 (1–11)	61 (16–109)	0.128
BNP, N = 10/0^†^	63 (28–345)	-	-
NT-prBNP, N = 19/2^†^	357.00 (92.10–2625.00)	3975.50 (909.00–7042.00)	0.400
*Immune-related, N = 23/4*^†^			
IgA, N = 21/4^†^	1.99 (1.47–2.43)	1.95 (1.32–2.36)	0.858
IgG, N = 21/4^†^	9.91 (8.29–12.46)	9.23 (6.51–17.09)	0.915
IgM, N = 20/4^†^	0.73 (0.43–1.47)	0.72 (0.48–2.01)	0.852
C3, N = 21/4^†^	1.12 (0.77–1.24)	0.47 (0.34–0.71)	0.003**
C4, N = 21/4^†^	0.28 (0.20–0.43)	0.30 (0.17–0.33)	0.620
Complement deficiency, N = 21/4^†^	3 (14.3)	3 (75.0)	0.031*
Anti-dsDNA antibodies, N = 20/4^†^	5 (25.0)	2 (50.0)	0.328
*Cell composition, N = 21/2*^†^			
CD3 + T cells (%), N = 20/2^†^	74.0 (61.3–86.0)	28.6 (0.1–57.1)	0.035*
CD4 + T cells (%), N = 20/2^†^	32.1 (27.5–36.6)	19.1 (14.6–23.6)	0.052
CD8 + T cells (%), N = 20/2^†^	41.5 (29.2–54.4)	49.2 (31.2–67.1)	0.701
CD4 + T cells/CD8 + T cells, N = 20/2^†^	0.78 (0.63–1.19)	0.49 (0.22–0.76)	0.312
CD3-CD19 + B cells (%), N = 20/2^†^	7.8 (2.4–16.6)	22.7 (15.4–29.9)	0.173
CD3-CD56 + CD16 + NK cells (%), N = 20/2^†^	14.4 (7.8–21.7)	49.0 (3.9–94.0)	0.779
T lymphocyte, N = 17/2^†^	228 (189–627)	301 (105–497)	0.749
CD4 + T lymphocyte, N = 17/2^†^	136 (76–183)	112 (19–205)	0.749
CD8 + T lymphocyte, N = 17/2^†^	125 (95–411)	179 (87–271)	0.842
B lymphocyte, N = 17/2^†^	23 (8–70)	140 (20–260)	0.351
NK cell, N = 17/2^†^	53 (33–72)	50 (5–94)	0.842
*Medication administration, N (%)*			
Glucocorticoids	29 (100.0)	5 (100.0)	–
Hydroxychloroquine	13 (44.8)	4 (80.0)	0.168
Immunosuppressants	7 (24.1)	4 (80.0)	0.029*
Antiviral	21 (72.4)	2 (40.0)	0.179
Antibiotics	19 (65.5)	4 (80.0)	0.471
Antipyretic analgesics	6 (20.7)	0	0.353
Traditional Chinese medicine preparations	8 (27.6)	1 (20.0)	0.600

^†^N = a/b indicates that this examination came from a) relieved SLE–COVID-19 comorbidity patients and b) worsened comorbidity patients

^*^, P < 0.05; ^**^, P < 0.01; ^***^, P < 0.001

### COVID-19 and SLE share hub gene signature enriched in IFN-I/II-related signaling pathway

**Fig. 1:**
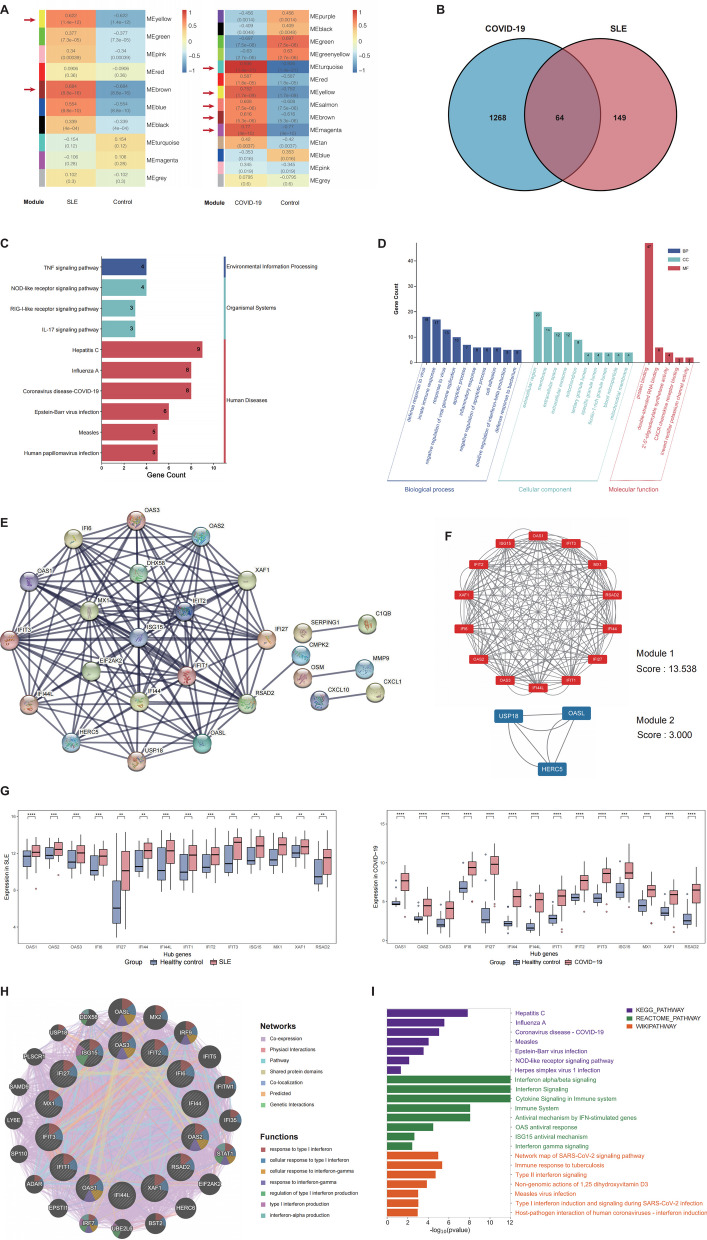
Hub genes of COVID-19 and SLE enriched in IFN-I/II-related signature. **A** Heatmap showing correlation coefficients and P-values of comparisons between clinical traits (COVID-19, SLE, and Control) and gene modules in the weighted gene co-expression network analysis. Modules with a correlation coefficient > 0.6 and P-value < 0.05 were identified as key gene modules (indicated by the red arrows in the figure); **B** Venn diagram showing the 64 common genes obtained after intersecting the key gene modules for SLE and COVID-19; **C** KEGG enrichment for common genes (P-value < 0.05); **D** GO term enrichment for common genes (P-value < 0.05); **E** PPI network for common genes at the highest confidence, which shows significant interactions between genes. The connecting line indicates the presence of an interaction, and the thickness of the line represents the confidence level, with a thicker line indicating higher confidence; **F** submodules 1 and 2 extracted from MCODE; **G** boxplots illustrating the variations in the expression levels of hub genes in SLE and COVID-19, where dots represent outliers (*P < 0.05, **P < 0.01, ***P < 0.001, ****P < 0.0001); **H** co-expression network for hub genes showing the IFN-related enrichment results with the lowest FDR; **I** pathway enrichment analysis for hub genes from KEGG, WIKI, and REACTOME databases (P-value < 0.05). *ME* module eigengene, *BP* biological process, *CC* cellular component, *MF* molecular function, *FDR* false discovery rate

To identify a shared disease-associated gene signature that elucidates the interconnection between COVID-19 and SLE, we focused on peripheral blood (PB), which is the main site for immune responses in COVID-19 and SLE, aside from the involved organs. As key components of PB, peripheral blood mononuclear cells (PBMCs) are also the main immune cells in the body responsible for initiating the immune response (Theofilopoulos et al. 2017). Therefore, we initially performed weighted gene co-expression network analysis on bulk RNA-seq datasets of PBMC samples from patients with SLE and COVID-19 (Additional file 1: Table S1) and obtained key gene modules associated with them (p-value < 0.05, correlation coefficient > 0.6) (Fig. 1A and Additional file 1: Figure S2). By intersecting genes from these key modules, we obtained 64 common genes that were significantly positively correlated with both diseases (Fig. 1B). KEGG analysis of these common genes revealed enrichment in infectious diseases, including COVID-19, Hepatitis C, and influenza A (Fig. 1C). Moreover, GO biological process analysis identified that these common genes predominantly participated in the immune response. GO molecular function analysis suggested a predominant association with protein binding (Fig. 1D), indicating that the pathogenic features shared between COVID-19 and SLE might be closely tied to the protein binding functionality of these common genes.

Subsequently, utilizing the STRING database, we constructed a PPI network of the common genes. Using the highest confidence criterion, we identified 26 genes that demonstrated significant interaction relevancy (Fig. 1E and Additional file 1: Figure S3). Using the MCODE algorithm, these 26 genes were further clustered into two submodules, with the score of submodule 1 substantially surpassing that of submodule 2 (Fig. 1F), suggesting that the 14 genes composing submodule 1 may serve as critical connector hub genes for constructing the PPI network of common genes. Additionally, the expression levels of these hub genes in COVID-19 and SLE groups were significantly elevated compared to those in the HC group (Fig. 1G). Further analysis with the GeneMANIA database revealed a strong co-expression between these hub genes and the genes they potentially interact with. GO analysis identified most genes related to viral and immune responses, encompassing cellular response to IFN-I/II (primarily IFN-α/β/γ) and regulation of IFN-I/II production pathways (Fig. 1H) (Additional file 1: Table S6). Moreover, pathway analysis of hub genes also significantly correlated with IFN signaling and cytokine signaling in the immune system (Fig. 1I). These findings suggest that co-expression interactions shared among hub genes between COVID-19 and SLE might be associated with IFN-I/II-related pathways.

### ML model identifies high diagnostic efficiency of hub genes in COVID-19 and SLE

**Fig. 2:**
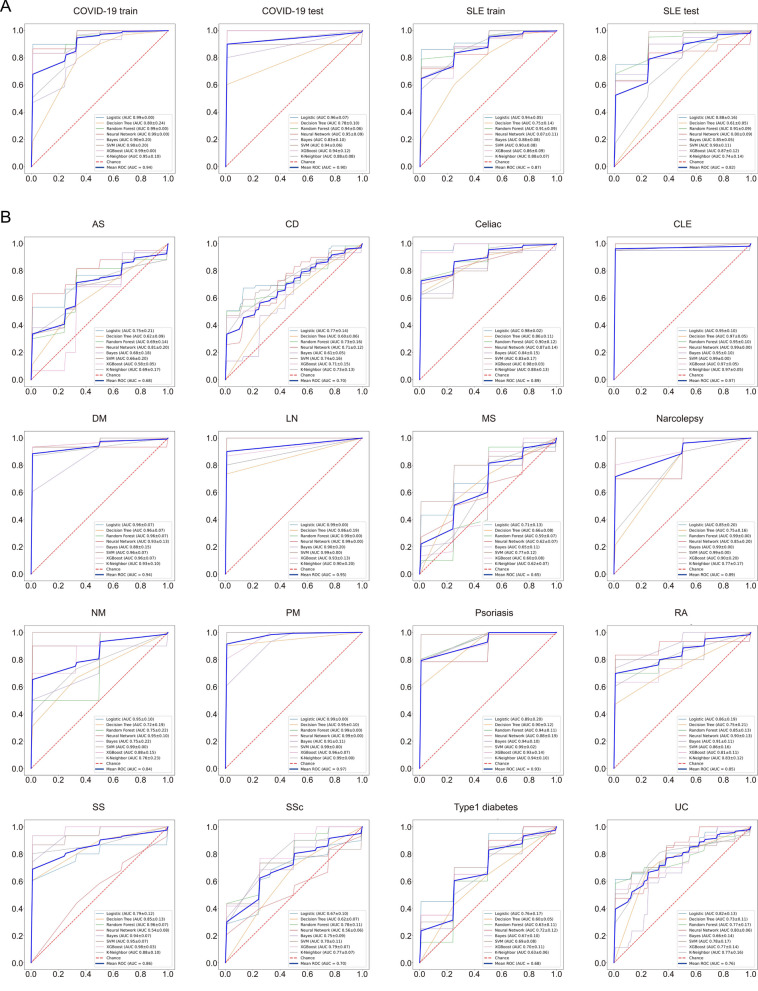
Efficiency of ML diagnostic model based on hub genes **A** Diagnostic efficiency of eight ML models on COVID-19 and SLE training or test datasets; **B** Diagnostic efficiency of eight ML models in pan-ADs. *AS* ankylosing spondylitis, *CD* Crohn's disease, *CLE* cutaneous lupus erythematosus, *DM* dermatomyositis, *LN* lupus nephritis, *MS* multiple sclerosis, *NM* necrotizing myopathy, *PM* polymyositis, *RA* rheumatoid arthritis, *SS* Sjogren's syndrome, *SSc* systemic sclerosis, *UC* ulcerative colitis

The ML method allows for more accurate and convenient clinical data collection and disease-prediction model construction (Hu et al. 2024). However, due to significant differences in the explanatory power of different ML models in different diseases (Hu et al. 2024), in order to comprehensively evaluate the diagnostic predictive capability of hub genes for COVID-19 and SLE, we built eight major ML models using the training datasets from both COVID-19 and SLE to establish a diagnostic prediction model. This model was designed to reflect the association of hub genes with the diseases based on their gene expression levels. Subsequently, we validated the model using the test datasets of bulk RNA-seq from both COVID-19 and SLE (Additional file 1: Table S1). The results demonstrated that the eight models constructed based on hub gene expression had an area under curve (AUC) value greater than 0.8 in their ROC curves for COVID-19 and SLE, with several models even achieving an AUC above 0.9 (Fig. 2A). This finding underscores the high diagnostic efficacy of hub genes for both COVID-19 and SLE. Furthermore, given the impressive predictive capacity of the diagnostic model in COVID-19 and SLE, we extended its application to 16 prevalent ADs, using their respective bulk RNA-seq datasets, to assess the diagnostic predictive performance of the model for pan-ADs. The diagnostic model exhibited an average AUC value above 0.6 in the ROC curve across pan-ADs, with 11 ADs presenting an average AUC value exceeding 0.8 (Fig. 2B). Such findings suggest that the diagnostic prediction model possesses significant diagnostic efficacy across a broad spectrum of ADs.

### Hub gene-related core TFs reveal JAK-STAT pathway activation in response to IFN-I/II

**Fig. 3:**
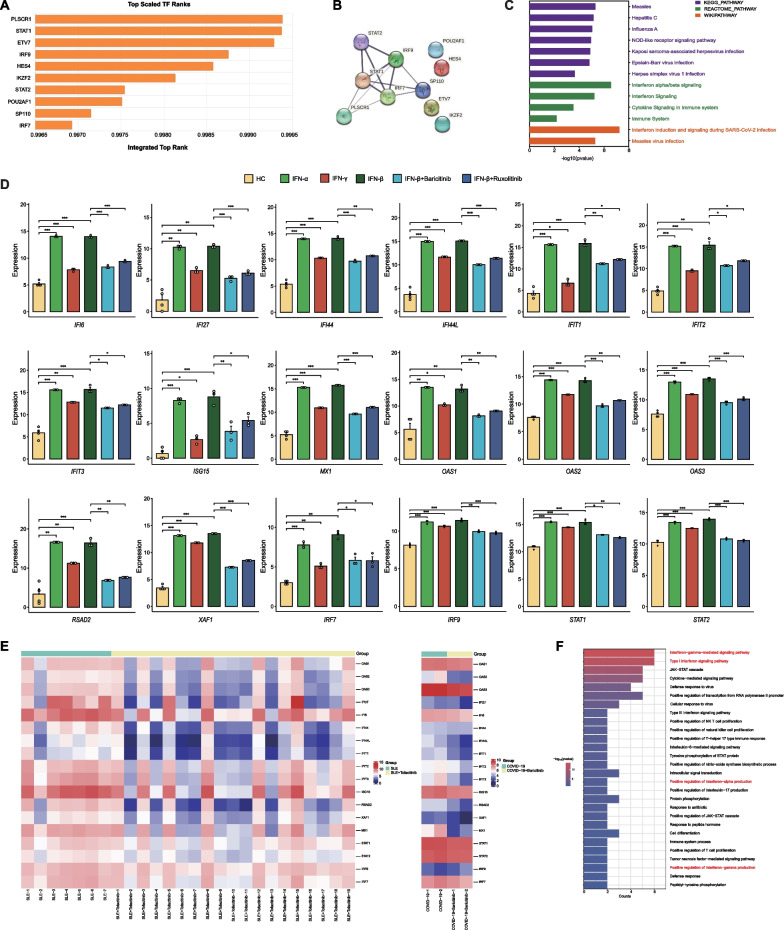
Hub genes and core TFs regulated by the JAK-STAT pathway **A** The top 10 TFs related to hub genes obtained from ChEA3 according to the integrated scaled rank; **B** PPI network of core TFs with significant interaction associations; **C** pathway enrichment analysis of core TFs from KEGG, WIKI, and REACTOME database (P-value < 0.05); **D** histograms showing the differences in the expression levels of hub genes and core TFs across six groups (*P < 0.05, **P < 0.01, ***P < 0.001). The six groups consisted of the HC group (samples from healthy people); the IFN-α, IFN-β, and IFN-γ groups (samples from healthy people treated with IFN-α, IFN-β, or IFN-γ); and the IFN-β + baricitinib or IFN-β + ruxolitinib groups (samples from healthy people treated with both JAK inhibitors, baricitinib or ruxolitinib, and IFN-β), respectively; **E** heatmap showing the changes in gene expression levels of hub genes and core TFs in SLE (left panel) or COVID-19 (right panel) after treatment with JAK inhibitors (tofacitinib or baricitinib); **F** Enrichment analysis of GO (BP) for JAKs (JAK1, JAK2, TYK2) and core TFs (P-value < 0.05). Terms in red font indicate enrichment results related to IFN-I/II

To further elucidate the regulatory molecular mechanism of the IFN-I/II-related signaling pathways on hub genes, we employed the ChIP-X Enrichment Analysis Version 3 database to identify the top 10 TFs closely associated with the transcriptional expression of hub genes (Fig. 3A). Subsequent STRING database analysis revealed significant interaction associations among four TFs: IRF7/9 and STAT 1/2 (Fig. 3B), which suggests that IRF7/9 and STAT1/2 are potentially the core TFs involved in regulating the expression of hub genes. Pathway analysis further validated the significant association of these core TFs with IFN induction and signaling during SARS-CoV-2 infection (Fig. 3C). Findings indicate that STAT1/2 are essential TFs of the IFN-I/II-triggered JAK-STAT signaling pathway, with IRF7 enhancing the activation of the pathway by promoting IFN-I/II production and IRF9 binding to STAT1/2 to activate nuclear IFN-related gene expression (Stark and Darnell 2012; Fernandez-Ruiz and Niewold 2022). Thus, the activation of the IFN-I/II triggered-JAK-STAT signaling pathway may predominantly regulate the transcriptional expression of hub genes in both COVID-19 and SLE.

To confirm the regulatory role of the JAK-STAT signaling pathway on hub genes, we compared the expression levels of hub genes and core TFs in six sample sets either treated or untreated with IFN-I/II (IFN-α/β/γ) or JAK-STAT signaling pathway inhibitors (baricitinib/ruxolitinib). We observed a consistent and significant trend in gene expression changes. Hub genes and core TFs exhibited elevated gene expression levels in the IFN-α/β and IFN-γ treatment groups compared with those in the HC group. In contrast, the expression levels of hub genes and core TFs were significantly lower in the IFN-β + baricitinib/ruxolitinib treatment groups than in the IFN-α/β groups, but higher than in the HC group, suggesting a simultaneous influence of IFN-α/β/γ and JAK inhibitors on hub genes and core TFs (Fig. 3D). These results affirm that hub genes are targets of the IFN-I/II-triggered JAK-STAT signaling pathway.

We then analyzed the expression level changes of hub genes and core TFs in SLE and COVID-19 samples after JAK inhibitor (tofacitinib or baricitinib) treatment, revealing a significant reduction in the expression of hub genes and core TFs (Fig. 3E), further proving that the JAK-STAT signaling pathway is crucial in regulating hub gene expression in both SLE and COVID-19. To assess the biological function of the hub genes related to the IFN-I/II-triggered JAK-STAT pathway, we conducted a GO analysis on the key components of the pathway (JAK1/2, Tyrosine kinase 2, and core TFs). The results were consistent with those from the enrichment analysis for hub genes, revealing a clear association with the regulation and production of IFN-I/II (Fig. 3F), which suggests that the hub gene-related JAK-STAT signaling pathway depends on the activation of IFN-I/II and is simultaneously related to IFN-I/II production. Building on these findings, we utilized the CMap database to further predict and identify the top 10 potential key molecular drugs targeting hub genes and their core TFs, including suloctidil, prasterone, propofol, and prochlorperazine, all of which displayed high relevancy (Additional file 1: Table S7).

### Crosstalk between COVID-19 and SLE highlights monokine production and CRS

**Fig. 4:**
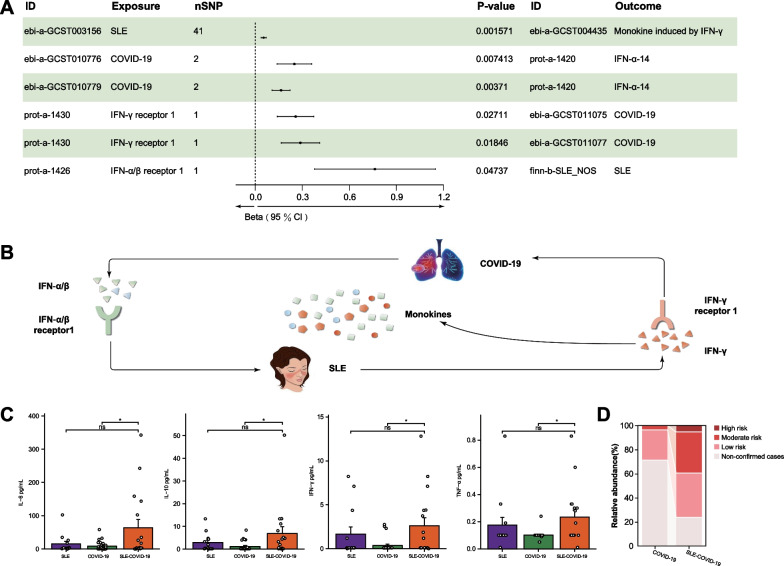
MR Analysis of crosstalk in SLE–COVID-19 **A** Forest plot for MR analysis results (P-value < 0.05) between SLE or COVID-19 and IFN-I/II-triggered JAK-STAT pathway-related molecules; **B** schematic diagram of MR analysis results illustrated in Figdraw; **C** comparison of serum monokines level between SLE, COVID-19, and SLE–COVID-19 (*P < 0.05). Error bars represent the standard error, available for 13 patients with SLE, 26 with COVID-19, and 17 with SLE–COVID-19. **D** Proportion of different risks of developing CRS in hospitalized COVID-19 and SLE–COVID-19 patients. *CI* Confidence interval

To further understand how the shared IFN-I/II-triggered JAK-STAT signaling pathway functions in the interplay between COVID-19 and SLE, we initially performed large-scale two-sample MR analysis designating either SLE or COVID-19 as exposure or outcome (Additional file 1: Table S2). All assessed GWAS datasets indicated no direct causal link between SLE and COVID-19 (Additional file 1: Table S8). Thus, we postulated a potential indirect mechanism facilitating their interaction. We then subjected the pivotal components of the IFN-I/II-triggered JAK-STAT pathway (IFN-I/II, JAKs, and core TFs) and the associated GWAS data for both SLE and COVID-19 diseases as exposure or outcome for another comprehensive two-sample MR analysis (Additional file 1: Table S2). Six analyses displayed significant causal connections (P < 0.05) (Fig. 4A and Additional file 1: Figure S4), and the MR insights revealed potential crosstalk between COVID-19 and SLE mediated through IFN-α/β receptor 1, IFN-γ receptor 1, IFN-α-14, and IFN-γ. Furthermore, this interaction culminated in the amplified release of IFN-γ-induced monokines (Fig. 4B).

To further elucidate the significance of monokines in the interaction between SLE and COVID-19, and considering that monokines, including IL-6, IL-10, TNF-α, and IFN-γ, have been linked to the onset and progression of both SLE and COVID-19 (Crow 2023; Liu, et al. 2022; Han et al. 2020), we subsequently analyzed the differences in levels of IL-6, IL-10, TNF-α, and IFN-γ between patients with SLE or COVID-19 and those with SLE–COVID-19 comorbidity. Our results revealed that compared with patients with COVID-19 alone, those with SLE–COVID-19 comorbidity exhibited significantly elevated levels of IL-6, IL-10, TNF-α, and IFN-γ (P < 0.05). However, when juxtaposed with patients with SLE, those with SLE–COVID-19 comorbidity displayed increased levels of these cytokines, but the increase was not significant (Fig. 4C). These findings suggest an augmented risk of monokine-related clinical symptoms in SLE–COVID-19 comorbidity.

Given the emerging understanding of the pivotal role of CRS in the pathogenesis and progression of COVID-19 (Moore and June 2020), we further compared the incidence and risk of CRS between COVID-19 and SLE–COVID-19 comorbidity (Memish et al. 2021). The incidence and risk of CRS in SLE–COVID-19 comorbid was significantly higher than those in COVID-19 alone (Fig. 4D), suggesting that the interplay between SLE and COVID-19 might exacerbate CRS occurrence in patients with comorbidity, potentially via enhancing monokine levels. Considering the multifaceted clinical manifestations of CRS, ranging from mild flu-like symptoms to severe overactive systemic immune responses leading to multi-organ failure (Shimabukuro-Vornhagen et al. 2018), we sought to further uncover the characteristics and impact of CRS in the context of SLE–COVID-19 interaction. By comparing the patients with SLE–COVID-19 comorbidity meeting the diagnostic criteria for CRS with the remainder, we discovered that non-CRS patients presented with elevated CRP, BNP, and NT-prBNP levels, but diminished WBC and monocyte counts and albumin levels (Additional file 1: Table S9), suggesting that CRS is primarily associated with hematological involvement, exacerbated inflammatory responses, and secondary organ damage, particularly cardiac damage. Such findings align with the main clinical features observed in patients with SLE–COVID-19 (Tables 1, 2, Additional file 1: Tables S3–5).

### Cellular expression signature of hub genes and core TFs further supported by the monocyte-macrophage

**Fig. 5:**
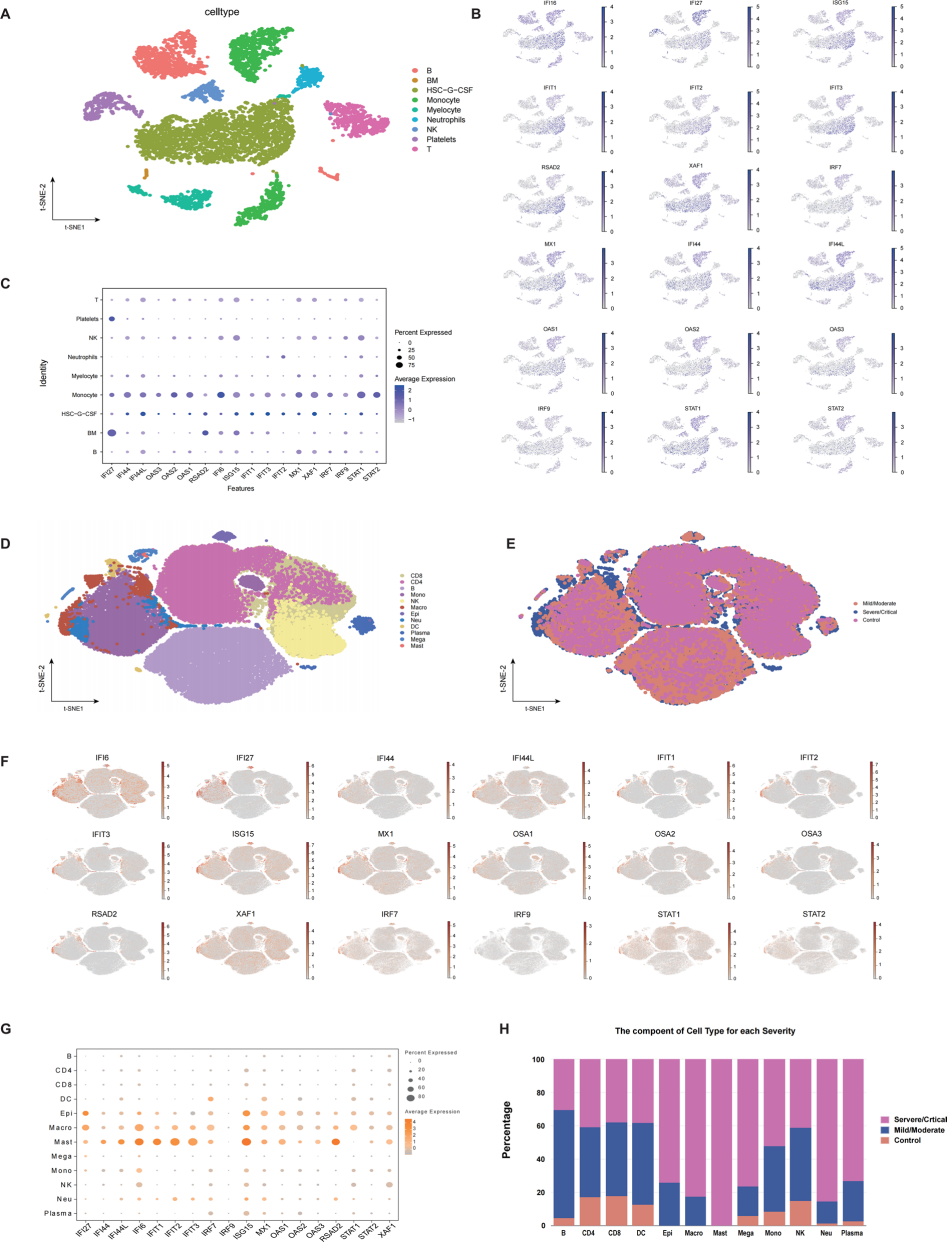
Cellular expression signatures of hub genes and core TFs across SLE and COVID-19 **A** The cell clustering result in SLE; **B**, **C** enriched expression of hub genes and core TFs in different cell clusters in SLE; **D**, **E** cell (**D**) and severity (**E**) clustering results in COVID-19; **F**, **G** enriched expressions of hub genes and core TFs in different cell clusters in COVID-19; **H** histograms showing the proportion of cell clustering in patients with COVID-19 at varying levels of disease severity. Each point in the scatter plot represents one cell, colored according to cell type, expression level, and severity; the size of each bubble represents the proportion of cells expressing the gene in the corresponding cell clustering while the depth of color indicates gene expression level

To further substantiate the pivotal role of monokines in the interaction between SLE and COVID-19, and considering that monokines are predominantly produced by monocytes-macrophages (Hume et al. 2019), we utilized the scRNA-seq datasets of PBMC samples from patients with SLE and COVID-19 to determine whether hub genes and core TFs are primarily enriched in monocytes/macrophages (Additional file 1: Table S4). In SLE, hub genes and core TFs were primarily expressed in monocytes and hematopoietic stem cells associated with granulocyte colony-stimulating factor production (Fig. 5A–C). In COVID-19, they were mainly expressed in macrophages, epithelial cells, and hypertrophic cells (Fig. 5D–G), with greater expression in patients with severe/critical COVID-19 (Fig. 5H). These findings indicate that core TFs and hub genes in both SLE and COVID-19 are chiefly expressed in monocytes/macrophages, corroborating the notion that monokines might play a significant role in the interplay between SLE and COVID-19. Moreover, the results emphasize the strong association between macrophages and severe/critical COVID-19.

## Discussion

**Fig. 6:**
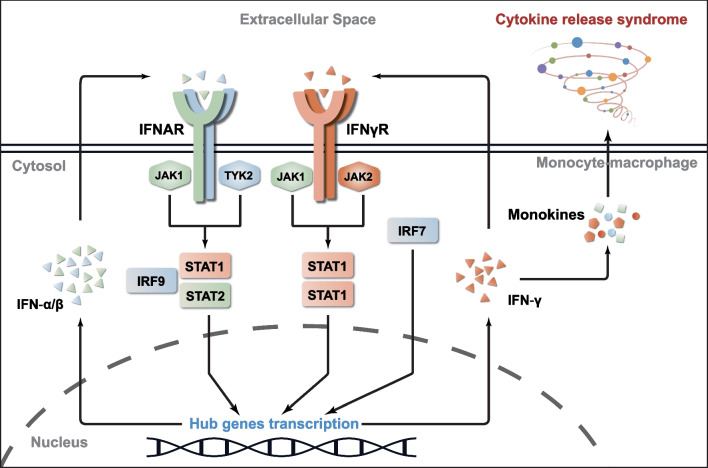
Schematic diagram of crosstalk between SLE and COVID-19. The solid lines with arrows represented direct regulation

The impact of COVID-19 on patients with SLE has not been extensively reported. From the analysis of clinical information from patients with SLE, SARS-CoV-2, COVID-19, SLE–SARS-CoV-2, and SLE–COVID-19 comorbidity, we revealed that hematological involvement and exacerbated inflammatory responses are the significant adverse effects of COVID-19 on patients with SLE. Additionally, transcriptomics- and genomics-related data revealed that the interaction between SLE and COVID-19 might be mediated by the IFN-I/II-triggered JAK-STAT signaling pathway, promoting the sustained production of monokines in patients with comorbidity, leading to CRS (Fig. 6). Thus, our results provide a scientific foundation for targeted diagnosis and treatment of patients with SLE–COVID-19 comorbidity.

Three conditions might explain the possible interaction between SLE and COVID-19: (1) Patients with SLE have a higher probability of contracting SARS-CoV-2 infection and later COVID-19; (2) SARS-CoV-2 infection increases the incidence of both COVID-19 and SLE; and (3) COVID-19 aggravates pathological status of SLE patients. Researchers in European countries thought that since SLE patients had been previously treated with corticoids, and/or immunosuppressants, they would be more vulnerable to SARS-CoV-2 infection and progression to COVID-19, which fits (1) (Cordtz, et al. 2021). As for (2), up to now, existing studies have observed an increase in the number of patients with COVID-19 during the pandemic (Pascolini et al. 2021), but no studies support the significant increase in the number of SLE patients. A study in Korea even found that the annual incidence rate of SLE in the population has declined (Ahn et al. 2023). Some studies have found a significant increase in hospitalization rates for SLE patients (Cordtz, et al. 2021; Schioppo et al. 2022) and that the prognostic risk and mortality rates of SLE patients with COVID-19 are significantly higher than those of the general population, supporting (3) (Bournia et al. 2023; Mageau et al. 2022). However, limited by the complex nature of underlying diseases in patients with SLE and the long-term use of corticosteroids (Kaul et al. 2016), a consensus on the adverse impact of COVID-19 on SLE is lacking. A study discovered that most risk factors leading to poor outcomes are not significantly different between patients with SLE–COVID-19 and the general population (Fernandez-Ruiz et al. 2021). Such reports indicate an urgent need for further research to elucidate the complex effects of COVID-19 on patients with SLE. Therefore, we comprehensively compared clinical and laboratory test results to examine the interaction between SLE and COVID-19. The SLE-DAI-2 K score and COVID-19-related clinical manifestations revealed no significant changes post-infection; however, we observed a tendency toward hematological involvement and exacerbated inflammatory responses (Kaul et al. 2016; Cordtz, et al. 2021). Therefore, our findings underscore the significance of the imbalanced immune response and over-activation of inflammatory responses shared by SLE and COVID-19 (Liu et al. 2021; Fernandez-Ruiz et al. 2021). However, considering that theory (1) suggested that patients with untreated SLE may exhibit a more robust immune response when complicated by SARS-CoV-2 infection (Schioppo et al. 2022), the results of our study may only be applicable to changes in clinical treatment strategies for patients with SLE–COVID-19, including the discontinuation of immunosuppressants and a switch to antiviral drugs in some patients, leading to an enhanced immune response. Additionally, patients with severely active SLE face poor prognoses after contracting COVID-19 (Bruera, et al. 2023). Our study also suggests that high SLE activity is closely related to disease exacerbation in patients with comorbidity, emphasizing the need for proactive SLE treatment in these patients. We also emphasize that secondary cardiac damage is another significant adverse effect of COVID-19 on patients with SLE, possibly closely related to the abnormal angiogenesis and impaired arterial tension commonly observed in them (Lai et al. 2021; Hasni et al. 2021; Ding et al. 2020; Casey, et al. 2021). In summary, our data suggest that clinical physicians must carefully consider patients with comorbidity with immunological disorders and provide active anti-inflammatory treatment.

Most patients with SLE exhibit high levels of IFN-I/II (Bastard, et al. 2020). While theoretically serving as an antiviral frontline, IFN-I/II may contribute to the excessive inflammatory response to COVID-19 (Fernandez-Ruiz et al. 2021). Furthermore, autoantibodies against IFN-α and IFN-ω have been detected during disease flare-ups in patients with life-threatening COVID-19 (Bastard et al. 2020). These studies suggest that IFN-I/II could be a pivotal bridge between SLE and COVID-19. In this study, we identified 14 IFN-I/II-related hub genes shared between SLE and COVID-19, emphasizing the crucial role of IFN-I/II in their shared pathogenic mechanisms. Notably, the expression of IFN-inducible (IFI) genes, such as *IFI27*, *IFI44*, *IFI44L*, *IFI6*, *IFIT1*, *IFIT2*, *IFIT3*, and *ISG15*, were correlated with disease activity and overexpression of autoimmune antibodies in SLE (Zhao et al. 2021), and associated with neutrophil-induced immunological imbalance and vascular damage (Mistry et al. 2019). In COVID-19, they are linked with the antiviral response and demonstrate consistent trends of enhanced inflammatory reactions in patients with severe COVID-19 (Gao et al. 2021; Wang et al. 2022; Alberts et al. 2022; Guo et al. 2022). The 2’-5’ oligoadenylate synthetase (OAS) gene family, including *OAS1, OAS2,* and *OAS3,* are closely related to the IFN-I/II-mediated signaling pathway in SLE (Gao et al. 2020) and are also tightly linked with the inflammatory response and production of IFN-I/II in COVID-19 (Lee, et al. 2023; Bruchelt et al. 2022). They might also be essential mediators in concurrent heart failure and cardiac injury in patients with COVID-19 (Gao et al. 2023). Similar to previous reports, in this study, the hub genes were significantly enriched in the IFN-I/II-related signaling pathway. Moreover, we unveiled a tight co-expression relationship among these hub genes for the first time. Other studies have revealed shared hub genes between COVID-19 and primary Sjogren's syndrome (Luo and Zhou 2022) or rheumatoid arthritis (Hu et al. 2022), such as MX1 and RSAD2, consistent with this study, indicating that the identified hub genes and their co-expression relations might be promising biomarkers for pan-ADs and COVID-19.

Both COVID-19 and SLE are typical inflammation-mediated diseases that exhibit numerous similarities (Fernandez-Ruiz et al. 2021). Therefore, in the early stages of the COVID-19 pandemic, hydroxychloroquine, a commonly used immunosuppressive drug for SLE patients (Ponticelli and Moroni 2017), attracted widespread attention (Wang et al. 2020). Unfortunately, many subsequent studies have confirmed that it does not exhibit antiviral activity nor enhance clinical therapeutic efficacy in patients (Maisonnasse et al. 2020; Hoffmann et al. 2020). Our research also indicates that compared with SLE patients, SLE–COVID-19 patients did not require additional treatment with hydroxychloroquine. However, in addition to JAK-STAT pathway inhibitor baricitinib, an emerging immunosuppressive treatment drug for SLE (Petri et al. 2023) has been approved by the FDA for clinical treatment of COVID-19 severe patients (Bronte et al. 2020). These results indicate that there are similar but complex and unknown immune inflammatory pathogeneses between SLE and COVID-19. In this context, considering that TFs act as key components in gene transcription regulation, their intricate regulatory networks often play a vital role in revealing the upstream regulatory mechanisms of target genes (Lambert et al. 2018). Here, by analyzing the core TFs related to hub genes, we demonstrated that IFN-I/II can modulate hub genes by activating the JAK-STAT signaling pathway. The JAK-STAT pathway has been implicated in the pathogenesis of various diseases, including ADs, infections, and cancers (Philips et al. 2022). In recent years, the significance of the JAK-STAT pathway in the progression of COVID-19 and SLE has been further elucidated (Philips et al. 2022). However, baricitinib can attenuate autoimmune functions in SLE mice, including kidney inflammation and podocyte protein structural anomalies (Lee et al. 2021). Additionally, the JAK/STAT pathway has been identified as an early promoter of COVID-19 and receptor binding (Shin et al. 2020). These findings further underscore the importance of the JAK-STAT signaling pathway and its inhibitors in understanding shared pathogenic mechanisms and targeted treatments between SLE and COVID-19. However, current JAK inhibitors, such as tofacitinib and baricitinib, have revealed significant safety concerns in clinical trials and treatments for SLE and COVID-19, including increased risks of secondary cancers, thrombosis, and major adverse cardiovascular events after medication (Zhang et al. 2020; Benucci et al. 2022). Therefore, there is a pressing need for further research to identify novel JAK-STAT pathway inhibitor drugs for supplementation or replacement of clinical treatments for SLE and COVID-19. In this study, we identified several candidate drugs targeting hub genes. Among them, the antithrombotic drug suloctidil was highlighted as the top therapeutic candidate, possibly due to its targeting of widespread vascular thrombotic events associated with COVID-19 and SLE (Farge et al. 2022; Andreoli et al. 2017). The steroid hormone prasterone, a common immunomodulator in SLE, also effectively manages disease activity and is believed to be a key mediator in the anti-COVID-19 action of 5α-reductase inhibitors (Durcan and Petri 2016; Papadopoulos et al. 2022). Moreover, propofol, often used for intubation sedation in patients with COVID-19, exhibits direct anti-COVID-19 effects by inhibiting sigma-1 receptors in vitro. Propofol also indirectly suppresses inflammation and thrombosis formation (Wei et al. 2021; Lucchetta et al. 2020). The broad-spectrum antiemetic prochlorperazine has not yet been reported for SLE or COVID-19 treatment; however, its potential effects on common COVID-19 symptoms, such as nausea and vomiting, are evident. Additionally, the antiviral capabilities of prochlorperazine have been validated in inhibiting the hepatitis C virus and dengue virus (Kow and Hasan 2021). Other candidate drugs, despite receiving high scores, are not frontline medications currently, and their significant side effects and safety concerns persist (Dougherty 1966; Chung et al. 1988; Soto et al. 1993; Fagbemi et al. 1984; Heel et al. 1978; Gehlawat et al. 2013), necessitating further assessment of the efficacies of these drugs in patients with SLE–COVID-19 comorbidity.

Several MR studies conducted between SLE and COVID-19 have demonstrated no direct causal relationship or common genetic architecture between both conditions (Yao et al. 2023; Xu et al. 2023; Quan et al. 2023). Similarly, our large-scale two-sample MR analysis between SLE and COVID-19 also showed no significant causal link between these diseases. Some studies have observed that in childhood and adolescent ADs, such as Kawasaki disease, the exacerbation of the disease precedes the onset of COVID-19-related symptoms, which suggests that the impact of COVID-19 on ADs might not be a direct action, but an immune-mediated process (Sancho-Shimizu, et al. 2021). Thus, we hypothesized that shared key pathways between SLE and COVID-19 might serve as vital channels for their interaction. SLE and COVID-19 might indirectly interact via IFN-I/II and its receptors, promoting the production of monokines. IFN-α may induce SLE when treating patients with malignancies (Rönnblom et al. 1990), and erythroid mitochondrial retention can induce SLE pathogenesis through IFN-I (Caielli et al. 2021). Furthermore, pathways involving the secretion of IFN-II-related cytokines or chemokines are significantly associated with COVID-19 pathogenesis (Buszko et al. 2021). In conclusion, these findings provide rheumatologists with novel strategies for approaching SLE–COVID-19 comorbidity.

Moreover, we also revealed that the levels of monokines, including IL-6/10, TNF-α, and IFN-γ, were significantly elevated in patients with comorbidity. The elevated IL-6 levels are a biomarker for diagnosing COVID-19 (Buszko et al. 2021) and are the main driving factor for long-term inflammatory responses in patients with severe COVID-19 (Cheong et al. 2023). Furthermore, the excessive release of various monokines, with IL-6 as the core monokine, is the primary cause of multi-systemic injuries and fatal outcomes in patients with severe COVID-19 (Xiao et al. 2021). IL-6 also interacts closely with other monokines. For example, high levels of IFN-γ in patients with SLE can promote IL-6 production, which may be related to the production of autoimmune antibodies (Arkatkar et al. 2017). Similarly, TNF-α can also play a significant role in neuroinflammatory responses in patients with SLE by promoting the levels of IL-6 (Kong et al. 2019). Further, three other three types of monokines also play significant roles. For example, in patients with SLE, IFN-γ can induce mononuclear macrophages to polarize to the M1 phenotype, thereby displaying pro-inflammatory functions (Liu et al. 2022); TNF-α promotes NADPH oxidase 2 gene expression in monocyte-macrophages in patients with SLE, thereby participating in the signal transduction of cytokines (Müller-Calleja et al. 2017); and in COVID-19, TNF-α and IFN-γ can also synergistically induce the death of inflammatory cells and tissue damage (Karki et al. 2021). IL-10 is generally considered an anti-inflammatory cytokine; however, it also displays IFN-I-mediated pro-inflammatory features in patients with SLE and is related to the disease activity (Idborg and Oke 2021). Meanwhile, IL-10 and IL-6 can distinguish and predict COVID-19 severity (Han et al. 2020). We further ascertained that in SLE and COVID-19, both hub genes and core TFs are enriched in the expression within monocytes/macrophages. As essential immune system components, monocytes/macrophages function primarily in phagocytosing pathogens, and mediating and promoting immune and inflammatory responses. Moreover, the dysregulation of their functions and relative abundances is closely related to the onset and development of SLE and COVID-19 (Gracia-Hernandez et al. 2020). Monocytes and macrophages involved in the pro-inflammatory response in SLE have higher expression levels of IFN-α-related genes, indicating their involvement in the pathogenesis of SLE. The relative abundance of different monocyte and macrophage subsets is significantly related to reactivity to IFNAR signaling in patients with SLE (Han, et al. 2020). Monocytes and macrophages can participate in SLE pathogenesis through IFN signaling; however, gasdermin D (GSDMD)-mediated monocyte/macrophage pyroptosis directly participates in inflammation in SLE (Zhuang et al. 2022). Similarly, in COVID-19, monocytes can trigger GSDMD-mediated inflammatory cell death, leading to a significant release of pro-inflammatory cytokines (Junqueira et al. 2022). These findings all support the importance of monokines and monocyte/macrophages in the interaction between SLE and COVID-19.

Furthermore, we discovered that CRS occurrence, strongly associated with elevated monokine levels, is a significant outcome of the interaction between SLE and COVID-19. Factors such as influenza virus or SARS-CoV-2 infections have been identified as triggers for CRS (Morris et al. 2022), and the substantial role of CRS in COVID-19 pathogenesis has become increasingly clear (Hu and Cho 2022). CRS is directly associated with tissue damage and adverse outcomes in patients with COVID-19 (Karki et al. 2021). The excessive activation of the immune response due to SARS-CoV-2 infection, resulting in CRS, is the direct cause of death in these patients (Vora et al. 2021). Patients with SLE undergoing long-term immunosuppressive therapy are at a higher risk of developing CRS, which may lead to severe organ failure in the presence of concurrent COVID-19 (Joob and Wiwanitkit 2020). This evidence underscores the crucial relationship between CRS and comorbidity. Additionally, patients with comorbid CRS are at elevated risk for cardiac impairment. CRS induced by COVID-19 frequently results in acute multi-organ injuries, including the myocardium (Ahmadian et al. 2021). Furthermore, cardiovascular system damage is often a predominant cause of severe outcomes in patients with SLE (Kaul et al. 2016). Overall, these findings should alert clinicians to the critical nature of immune dysregulation associated with CRS and the consequent cardiac impairment in patients with comorbidity while also indicating potential targeted treatment strategies based on the interaction mechanisms between SLE and COVID-19.

Our study also has some limitations. First, despite incorporating as many GWAS datasets as possible from the IEU database that satisfy our study criteria, only one group had sufficient SNPs and passed the horizontal pleiotropy test in the MR analysis (Additional file 1: Figure S4). The GWAS data related to larger scales of SLE, COVID-19, and IFN-I/II should be updated. Second, considering the gender differences in inflammatory response (Pan and Chang 2012), the number of male SLE cases included in this study is limited. Additionally, due to the limited sample size, the comparative analysis between COVID-19 and COVID-19 + Baricitinib lacks enough notorious changes. Third, the detection of relevant cytokines was conducted only in a subset of patients, which limited our ability to comprehensively analyze the complex changes in monokine levels and the clinical characteristics of CRS in patients with comorbidity. More importantly, whether these observations are reproducible in larger cohorts requires investigation.

## Conclusions

The interaction between SLE and COVID-19 promotes the activation of the IFN-I/II-triggered JAK-STAT signaling pathway in monocytes/macrophages, which elevates the levels of IL-6/10, TNF-α, and IFN-γ, and increases the incidence and risk of CRS in patients with SLE–COVID-19 comorbidity. These findings provide a new direction and rationale for diagnosing and treating patients with SLE–COVID-19 comorbidity (Additional file 1: Figure S5).

### Supplementary Information

**Additional file 1: Supplementary Figure 1.** Quality control of raw count matrix in scRNA-seq of SLE.** Supplementary Figure 2.** Soft threshold power in WGCNA of bulk RNA-seq datasets from patients with SLE and COVID-19.** Supplementary Figure 3.** PPI network of common genes with medium confidence.** Supplementary Figure 4.** Results of Leave-one-out, MR-Egger intercept, and Cochran’s Q analyses for MR sensitivity, pleiotropy, and heterogeneity testing.** Supplementary Figure 5.** Cover figure of the manuscript.** Supplementary Table 1.** RNA-seq datasets from GEO.** Supplementary Table 2.** GWAS datasets from IEU OpenGWAS project.** Supplementary Table 3.** Clinical information comparison between SLE and SARS-CoV-2-infected SLE outpatients.** Supplementary Table 4.** Clinical information comparison before and after contracting COVID-19 in hospitalized SLE patients (paired samples).** Supplementary Table 5.** Clinical information comparison between SLE outpatients with mild and moderate/severe SARS-CoV-2 infection.** Supplementary Table 6.** GO analysis of hub genes from GeneMANIA.** Supplementary Table 7.** Top 10 drug signature of hub genes and core TFs according to combined score from CMAP.** Supplementary Table 8.** Results of MR analysis between SLE and COVID-19.** Supplementary Table 9.** Clinical information comparison between hospitalized SLE–COVID-19 patients diagnosed as CRS and non-CRS.

## Data Availability

Data will be made available on request.

## Abbreviations

COVID-19: Coronavirus disease 2019
SARS-CoV-2: Severe acute respiratory syndrome coronavirus 2
ADs: Autoimmune diseases
SLE: Systemic lupus erythematosus
IL: Interleukin
TNF-α: Tumor necrosis factor α
IFN-α: Interferon α
RNA-seq: RNA sequencing
PBMCs: Peripheral blood mononuclear cells
ML: Machine learning
TFs: Transcription factors
GWAS: Genome-wide association study
scRNA-seq: Single-cell RNA-seq
SLEDAI-2K: SLE Disease Activity Index 2000
CRS: Cytokine release syndrome
CRP: C-reactive protein
WGCNA: Weighted correlation network analysis
JAK-STAT: Janus kinase/signal transducers and activators of transcription
IRFs: IFN regulatory factors
SNPs: Single nucleotide polymorphisms
MAD: Maximum mean absolute deviation
STRING: The Search Tool for the Retrieval of Interacting Genes
PPI: Protein–protein interaction
MCODE: Molecular Complex Detection
GO: Gene Ontology
DAVID: Database for Annotation, Visualization, and Integrated Discovery
KEGG: Kyoto Encyclopedia of Genes and Genomes
CMap: Connectivity Map
ROC: Receiver operating characteristic
MR: Mendelian randomization
LD: Linkage disequilibrium
IVW: Inverse variance weighted
TSNE: T-distributed stochastic neighbor embedding
SPSS: Statistical Product and Service Solutions
PLC: Platelet count
WBC: White blood cell
NT-prBNP: N-terminal pro-B-type natriuretic peptide
PB: Peripheral blood
HCs: Healthy controls
AUC: Area under curve
BNP: B-type natriuretic peptide
IFI: IFN-inducible
ISG: Interferon-stimulated gene
OAS: Oligoadenylate synthetase
GSDMD: Gasdermin D
RBC: Red blood cell
HGB: Hemoglobin
AEC: Absolute eosinophil count
ANC: Absolute neutrophil count
AMC: Absolute monocyte count
ABC: Absolute basophil count
ESR: Erythrocyte sedimentation rate
ALT: Alanine transaminase
AST: Aspartate transaminase
ALP: Alkaline phosphatase
GGT: Gamma-glutamyl transferase
HDL-C: High density lipoprotein cholesterol
LDL-C: Low density lipoprotein cholesterol
LEU: Urinary leukocyte count
IgA: Immunoglobulin A
IgG: Immunoglobulin G
IgM: Immunoglobulin M
C3: Complement 3
C4: Complement 4
NK: Natural killer

## References

[CR1] Ahmadian E (2021). Covid-19 and kidney injury: pathophysiology and molecular mechanisms. Rev Med Virol.

[CR2] Ahn SM (2023). Incidence of rheumatic diseases during the COVID-19 pandemic in South Korea. Korean J Intern Med.

[CR3] Alberts R (2022). Integration and reanalysis of four RNA-Seq datasets including BALF, nasopharyngeal swabs, lung biopsy, and mouse models reveals common immune features of COVID-19. Immune Network.

[CR4] Andreoli L (2017). EULAR recommendations for women's health and the management of family planning, assisted reproduction, pregnancy and menopause in patients with systemic lupus erythematosus and/or antiphospholipid syndrome. Ann Rheum Dis.

[CR5] Arkatkar T (2017). B cell-derived IL-6 initiates spontaneous germinal center formation during systemic autoimmunity. J Exp Med.

[CR6] Bader GD, Hogue CW (2003). An automated method for finding molecular complexes in large protein interaction networks. BMC Bioinform.

[CR7] Barrett T (2011). NCBI GEO: archive for functional genomics data sets–10 years on. Nucleic Acids Res.

[CR8] Bastard P, et al. Autoantibodies against type I IFNs in patients with life-threatening COVID-19. Science (New York, NY). 2020; 370. 10.1126/science.abd4585 PMC7857397 32972996

[CR9] Benucci M (2022). Cardiovascular safety, cancer and Jak-inhibitors: Differences to be highlighted. Pharmacol Res.

[CR10] Bonometti R (2020). The first case of systemic lupus erythematosus (SLE) triggered by COVID-19 infection. Eur Rev Med Pharmacol Sci.

[CR11] Bournia VK (2023). Different COVID-19 outcomes among systemic rheumatic diseases: a nation-wide cohort study. Rheumatology (oxford).

[CR12] Bronte V (2020). Baricitinib restrains the immune dysregulation in patients with severe COVID-19. J Clin Investig.

[CR13] Bruchelt G, Treuner J, Schmidt K (2022). Proposal for the use of an inhalation drug containing 2–5 oligoadenylates for treatment of COVID-19. Med Hypotheses.

[CR14] Bruera S et al. Risks of mortality and severe coronavirus disease 19 (COVID-19) outcomes in patients with or without systemic lupus erythematosus. Lupus Sci Med. 2023; 10. 10.1136/lupus-2022-000750 PMC9929928 36787921

[CR15] Buszko M (2021). Lessons learned: new insights on the role of cytokines in COVID-19. Nat Immunol.

[CR16] Caielli S (2021). Erythroid mitochondrial retention triggers myeloid-dependent type I interferon in human SLE. Cell.

[CR17] Casey KA (2021). Modulation of cardiometabolic disease markers by type I interferon inhibition in systemic lupus erythematosus. Arthritis Rheumatol (hoboken, NJ).

[CR18] Chen T, Liu Y-X, Huang L (2022). ImageGP: an easy-to-use data visualization web server for scientific researchers. iMeta.

[CR19] Cheong JG (2023). Epigenetic memory of coronavirus infection in innate immune cells and their progenitors. Cell.

[CR20] Chung MW, Komorowski RA, Varma RR (1988). Suloctidil-induced hepatotoxicity. Gastroenterology.

[CR21] Cordtz R et al. Incidence of COVID-19 hospitalisation in patients with systemic lupus erythematosus: a nationwide cohort study from Denmark. J Clin Med. 2021; 10. 10.3390/jcm10173842 PMC8432052 34501290

[CR22] Crow MK (2023). Pathogenesis of systemic lupus erythematosus: risks, mechanisms and therapeutic targets. Ann Rheum Dis.

[CR23] da Huang W, Sherman BT, Lempicki RA (2009). Systematic and integrative analysis of large gene lists using DAVID bioinformatics resources. Nat Protoc.

[CR24] Ding X, Xiang W, He X (2020). IFN-I mediates dysfunction of endothelial progenitor cells in atherosclerosis of systemic lupus erythematosus. Front Immunol.

[CR25] Dougherty J (1966). Hypoglycemic stupor caused by acetohexamide. N Engl J Med.

[CR26] Durcan L, Petri M (2016). Immunomodulators in SLE: clinical evidence and immunologic actions. J Autoimmun.

[CR27] Fagbemi O, Kane KA, McDonald FM, Parratt JR, Rothaul AL (1984). The effects of verapamil, prenylamine, flunarizine and cinnarizine on coronary artery occlusion-induced arrhythmias in anaesthetized rats. Br J Pharmacol.

[CR28] Fanouriakis A, Tziolos N, Bertsias G, Boumpas DT (2021). Update οn the diagnosis and management of systemic lupus erythematosus. Ann Rheum Dis.

[CR29] Fanouriakis A (2024). EULAR recommendations for the management of systemic lupus erythematosus: 2023 update. Ann Rheum Dis.

[CR30] Farge D (2022). 2022 international clinical practice guidelines for the treatment and prophylaxis of venous thromboembolism in patients with cancer, including patients with COVID-19. Lancet Oncol.

[CR31] Fernandez-Ruiz R, Niewold TB (2022). Type I interferons in autoimmunity. J Invest Dermatol.

[CR32] Fernandez-Ruiz R, Paredes JL, Niewold TB (2021). COVID-19 in patients with systemic lupus erythematosus: lessons learned from the inflammatory disease. Transl Res J Lab Clin Med.

[CR33] Ferri C (2020). COVID-19 and rheumatic autoimmune systemic diseases: report of a large Italian patients series. Clin Rheumatol.

[CR34] Franz M (2018). GeneMANIA update 2018. Nucleic Acids Res.

[CR35] Gao F, Tan Y, Luo H (2020). MALAT1 is involved in type I IFNs-mediated systemic lupus erythematosus by up-regulating OAS2, OAS3, and OASL. Braz J Md Biol Res Revista Brasileira De Pesquisas Medicas e Biologicas.

[CR36] Gao X (2021). Genome-wide screening of SARS-CoV-2 infection-related genes based on the blood leukocytes sequencing data set of patients with COVID-19. J Med Virol.

[CR37] Gao LJ (2023). Role of OAS gene family in COVID-19 induced heart failure. J Transl Med.

[CR38] Garrido I (2021). Autoimmune hepatitis after COVID-19 vaccine—more than a coincidence. J Autoimmun.

[CR39] Gehlawat P, Singh P, Gupta R, Arya S (2013). Mephentermine dependence with psychosis. Gen Hosp Psychiatry.

[CR40] Gracia-Hernandez M, Sotomayor EM, Villagra A (2020). Targeting macrophages as a therapeutic option in coronavirus disease 2019. Front Pharmacol.

[CR41] Guo C (2022). Single-cell transcriptomics reveal a unique memory-like NK cell subset that accumulates with ageing and correlates with disease severity in COVID-19. Genome Med.

[CR42] Han H (2020). Profiling serum cytokines in COVID-19 patients reveals IL-6 and IL-10 are disease severity predictors. Emerg Microbes Infect.

[CR43] Han S (2020). Differential responsiveness of monocyte and macrophage subsets to interferon. Arthritis Rheumatol (hoboken, NJ).

[CR44] Hasni SA (2021). Phase 1 double-blind randomized safety trial of the Janus kinase inhibitor tofacitinib in systemic lupus erythematosus. Nat Commun.

[CR45] Heel RC, Brogden RN, Speight TM, Avery GS (1978). Econazole: a review of its antifungal activity and therapeutic efficacy. Drugs.

[CR46] Hoffmann M (2020). Chloroquine does not inhibit infection of human lung cells with SARS-CoV-2. Nature.

[CR47] Hu T, Cho CH (2022). Cytokine release syndrome in pathogenesis and treatment of COVID-19. Curr Pharm Des.

[CR48] Hu H, Tang N, Zhang F, Li L, Li L (2022). Bioinformatics and system biology approach to identify the influences of COVID-19 on rheumatoid arthritis. Front Immunol.

[CR49] Hu J (2024). Identification and validation of an explainable prediction model of acute kidney injury with prognostic implications in critically ill children: a prospective multicenter cohort study. EClinicalMedicine.

[CR50] Hume DA, Irvine KM, Pridans C (2019). The mononuclear phagocyte system: the relationship between monocytes and macrophages. Trends Immunol.

[CR51] Idborg H, Oke V. Cytokines as biomarkers in systemic lupus erythematosus: value for diagnosis and drug therapy. Int J Mol Sci. 2021; 22. 10.3390/ijms222111327 PMC8582965 34768756

[CR52] Joob B, Wiwanitkit V (2020). SLE, hydroxychloroquine and no SLE patients with COVID-19: a comment. Ann Rheum Dis.

[CR53] Junqueira C (2022). FcγR-mediated SARS-CoV-2 infection of monocytes activates inflammation. Nature.

[CR54] Karki R (2021). Synergism of TNF-α and IFN-γ triggers inflammatory cell death, tissue damage, and mortality in SARS-CoV-2 infection and cytokine shock syndromes. Cell.

[CR55] Kaul A (2016). Systemic lupus erythematosus. Nat Rev Dis Primers.

[CR56] Keenan AB (2019). ChEA3: transcription factor enrichment analysis by orthogonal omics integration. Nucleic Acids Res.

[CR57] Kong X (2019). TNF-α regulates microglial activation via the NF-κB signaling pathway in systemic lupus erythematosus with depression. Int J Biol Macromol.

[CR58] Kow CS, Hasan SS (2021). Prochlorperazine for nausea and vomiting accompanied COVID-19. J Gastroenterol Hepatol.

[CR59] Kuleshov MV (2016). Enrichr: a comprehensive gene set enrichment analysis web server 2016 update. Nucleic Acids Res.

[CR60] Lai JH (2021). Mitochondrial protein CMPK2 regulates IFN alpha-enhanced foam cell formation, potentially contributing to premature atherosclerosis in SLE. Arthritis Res Ther.

[CR61] Lambert SA (2018). The human transcription factors. Cell.

[CR62] Langfelder P, Horvath S (2008). WGCNA: an R package for weighted correlation network analysis. BMC Bioinform.

[CR63] Lee J (2021). Baricitinib attenuates autoimmune phenotype and podocyte injury in a murine model of systemic lupus erythematosus. Front Immunol.

[CR64] Lee D (2023). Inborn errors of OAS-RNase L in SARS-CoV-2-related multisystem inflammatory syndrome in children. Science (new York, NY).

[CR65] Liu Y, Sawalha AH, Lu Q (2021). COVID-19 and autoimmune diseases. Curr Opin Rheumatol.

[CR66] Liu W, Zhang S, Wang J (2022). IFN-γ, should not be ignored in SLE. Front Immunol.

[CR67] Liu X (2022). SARS-CoV-2 spike protein-induced cell fusion activates the cGAS-STING pathway and the interferon response. Sci Signal.

[CR68] Lowery SA, Sariol A, Perlman S (2021). Innate immune and inflammatory responses to SARS-CoV-2: implications for COVID-19. Cell Host Microbe.

[CR69] Lucchetta V, Bonvicini D, Ballin A, Tiberio I (2020). Propofol infusion syndrome in severe COVID-19. Br J Anaesth.

[CR70] Luo H, Zhou X (2022). Bioinformatics analysis of potential common pathogenic mechanisms for COVID-19 infection and primary Sjogren's syndrome. Front Immunol.

[CR71] Mageau A (2022). Survival after COVID-19-associated organ failure among inpatients with systemic lupus erythematosus in France: a nationwide study. Ann Rheum Dis.

[CR72] Maisonnasse P (2020). Hydroxychloroquine use against SARS-CoV-2 infection in non-human primates. Nature.

[CR73] Memish ZA, Faqihi F, Alharthy A, Alqahtani SA, Karakitsos D (2021). Plasma exchange in the treatment of complex COVID-19-related critical illness: controversies and perspectives. Int J Antimicrob Agents.

[CR74] Mistry P (2019). Transcriptomic, epigenetic, and functional analyses implicate neutrophil diversity in the pathogenesis of systemic lupus erythematosus. Proc Natl Acad Sci USA.

[CR75] Moore JB, June CH (2020). Cytokine release syndrome in severe COVID-19. Science (new York, NY).

[CR76] Morris EC, Neelapu SS, Giavridis T, Sadelain M (2022). Cytokine release syndrome and associated neurotoxicity in cancer immunotherapy. Nat Rev Immunol.

[CR77] Müller-Calleja N, Manukyan D, Canisius A, Strand D, Lackner KJ (2017). Hydroxychloroquine inhibits proinflammatory signalling pathways by targeting endosomal NADPH oxidase. Ann Rheum Dis.

[CR78] Number of COVID-19 deaths reported to WHO (cumulative total). 12 May 2024. https://data.who.int/dashboards/covid19/deaths?n=c.

[CR79] Pan Z, Chang C (2012). Gender and the regulation of longevity: implications for autoimmunity. Autoimmun Rev.

[CR80] Papadopoulos KI, Papadopoulou A, Sutheesophon W, Aw TC (2022). Anti-SARS-CoV-2 action of 5α-reductase inhibitors may be mediated by dehydroepiandrosterone. Lett J Urol.

[CR81] Pascolini S (2021). COVID-19 and immunological dysregulation: can autoantibodies be useful?. Clin Transl Sci.

[CR82] Petri M (2023). Baricitinib for systemic lupus erythematosus: a double-blind, randomised, placebo-controlled, phase 3 trial (SLE-BRAVE-II). Lancet (london, England).

[CR83] Philips RL (2022). The JAK-STAT pathway at 30: much learned, much more to do. Cell.

[CR84] Ponticelli C, Moroni G (2017). Hydroxychloroquine in systemic lupus erythematosus (SLE). Expert Opin Drug Saf.

[CR85] Quan L, Tan J, Hua L, You X (2023). Genetic predisposition between coronavirus disease 2019 and rheumatic diseases: a 2-sample Mendelian randomization study. Int J Rheum Dis.

[CR86] Rönnblom LE, Alm GV, Oberg KE (1990). Possible induction of systemic lupus erythematosus by interferon-alpha treatment in a patient with a malignant carcinoid tumour. J Intern Med.

[CR87] Sancho-Shimizu V, et al. SARS-CoV-2-related MIS-C: a key to the viral and genetic causes of Kawasaki disease? J Exp Med. 2021; 218. 10.1084/jem.20210446 PMC8080850 33904890

[CR88] Schioppo T (2022). Clinical and peculiar immunological manifestations of SARS-CoV-2 infection in systemic lupus erythematosus patients. Rheumatology (oxford).

[CR89] Shannon P (2003). Cytoscape: a software environment for integrated models of biomolecular interaction networks. Genome Res.

[CR90] Shimabukuro-Vornhagen A (2018). Cytokine release syndrome. J Immunother Cancer.

[CR91] Shin D (2020). Papain-like protease regulates SARS-CoV-2 viral spread and innate immunity. Nature.

[CR92] Soto J, Sacristan JA, Alsar MJ, Sainz C (1993). Terfenadine-induced tremor. Ann Neurol.

[CR93] Stark GR, Darnell JE (2012). The JAK-STAT pathway at twenty. Immunity.

[CR94] Su WM (2023). Systematic druggable genome-wide Mendelian randomisation identifies therapeutic targets for Alzheimer's disease. J Neurol Neurosurg Psychiatry.

[CR95] Szklarczyk D (2019). STRING v11: protein-protein association networks with increased coverage, supporting functional discovery in genome-wide experimental datasets. Nucleic Acids Res.

[CR96] Theofilopoulos AN, Kono DH, Baccala R (2017). The multiple pathways to autoimmunity. Nat Immunol.

[CR97] Vora SM, Lieberman J, Wu H (2021). Inflammasome activation at the crux of severe COVID-19. Nat Rev Immunol.

[CR98] Wang M (2020). Remdesivir and chloroquine effectively inhibit the recently emerged novel coronavirus (2019-nCoV) in vitro. Cell Res.

[CR99] Wang Y (2022). Plasma cell-free RNA characteristics in COVID-19 patients. Genome Res.

[CR100] Wei P (2021). Putative antiviral effects of propofol in COVID-19. Br J Anaesth.

[CR101] Xiao N (2021). Integrated cytokine and metabolite analysis reveals immunometabolic reprogramming in COVID-19 patients with therapeutic implications. Nat Commun.

[CR102] Xu SZ (2023). No genetic causal association between systemic lupus erythematosus and COVID-19. Front Immunol.

[CR103] Yao M, Huang X, Guo Y, Zhao JV, Liu Z (2023). Disentangling the common genetic architecture and causality of rheumatoid arthritis and systemic lupus erythematosus with COVID-19 outcomes: genome-wide cross trait analysis and bidirectional Mendelian randomization study. J Med Virol.

[CR104] Yoo M (2015). DSigDB: drug signatures database for gene set analysis. Bioinformatics (oxford, England).

[CR105] Zhang X, Zhang Y, Qiao W, Zhang J, Qi Z (2020). Baricitinib, a drug with potential effect to prevent SARS-COV-2 from entering target cells and control cytokine storm induced by COVID-19. Int Immunopharmacol.

[CR106] Zhao X (2021). Identification of key biomarkers and immune infiltration in systemic lupus erythematosus by integrated bioinformatics analysis. J Transl Med.

[CR107] Zhuang L (2022). Disulfiram alleviates pristane-induced lupus via inhibiting GSDMD-mediated pyroptosis. Cell Death Discov.

