# Supplementary material for: Multi-omics analysis uncovered systemic lupus erythematosus and COVID-19 crosstalk
Source: Mol Med. 2024 Jun 11;30:81. doi: 10.1186/s10020-024-00851-6 (PMC11167821; doi:10.1186/s10020-024-00851-6)
Supplement: Supplementary file 1 — Additional file 1: Supplementary Figure 1. Quality control of raw count matrix in scRNA-seq of SLE. Supplementary Figure 2. Soft threshold power in WGCNA of bulk RNA-seq datasets from patients with SLE and COVID-19. Supplementary Figure 3. PPI network of common genes with medium confidence. Supplementary Figure 4. Results of Leave-one-out, MR-Egger intercept, and Cochran’s Q analyses for MR sensitivity, pleiotropy, and heterogeneity testing. Supplementary Figure 5. Cover figure of the manuscript. Supplementary Table 1. RNA-seq datasets from GEO. Supplementary Table 2. GWAS datasets from IEU OpenGWAS project. Supplementary Table 3. Clinical information comparison between SLE and SARS-CoV-2-infected SLE outpatients. Supplementary Table 4. Clinical information comparison before and after contracting COVID-19 in hospitalized SLE patients (paired samples). Supplementary Table 5. Clinical information comparison between SLE outpatients with mild and moderate/severe SARS-CoV-2 infection. Supplementary Table 6. GO analysis of hub genes from GeneMANIA. Supplementary Table 7. Top 10 drug signature of hub genes and core TFs according to combined score from CMAP. Supplementary Table 8. Results of MR analysis between SLE and COVID-19. Supplementary Table 9. Clinical information comparison between hospitalized SLE–COVID-19 patients diagnosed as CRS and non-CRS. [file 10020_2024_851_MOESM1_ESM.docx]

**Multi-omics analysis uncovered systemic lupus erythematosus and COVID-19 crosstalk**

**ADDITIONAL FILE**

**Supplementary figures**

**
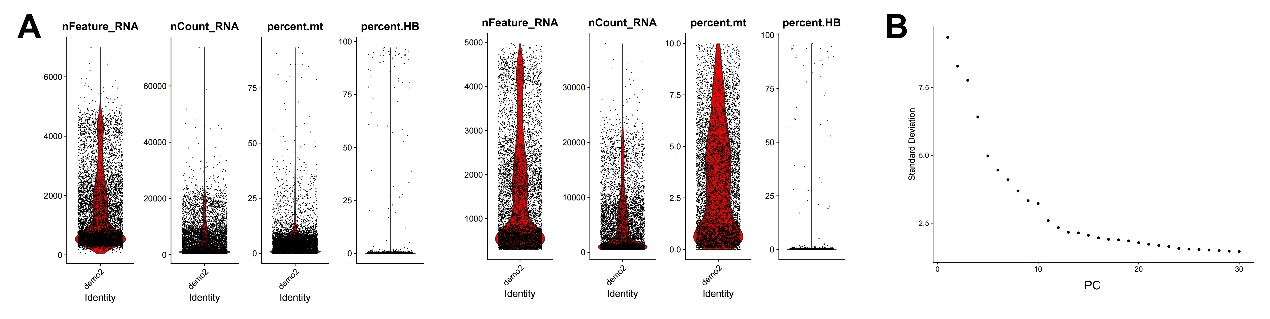
**

**Supplementary figure 1. Quality control of raw count matrix in scRNA-seq of SLE.** (A) Comparison before and after cell filtering based on the number of genes in each cell, total gene expression, percentage of mitochondrial genes, and percentage of red blood cell genes (Left panel: before filtering; Right panel: after filtering); (B) Scree plot showing the standard deviations corresponding to different numbers of principal components selected in PCA.

**
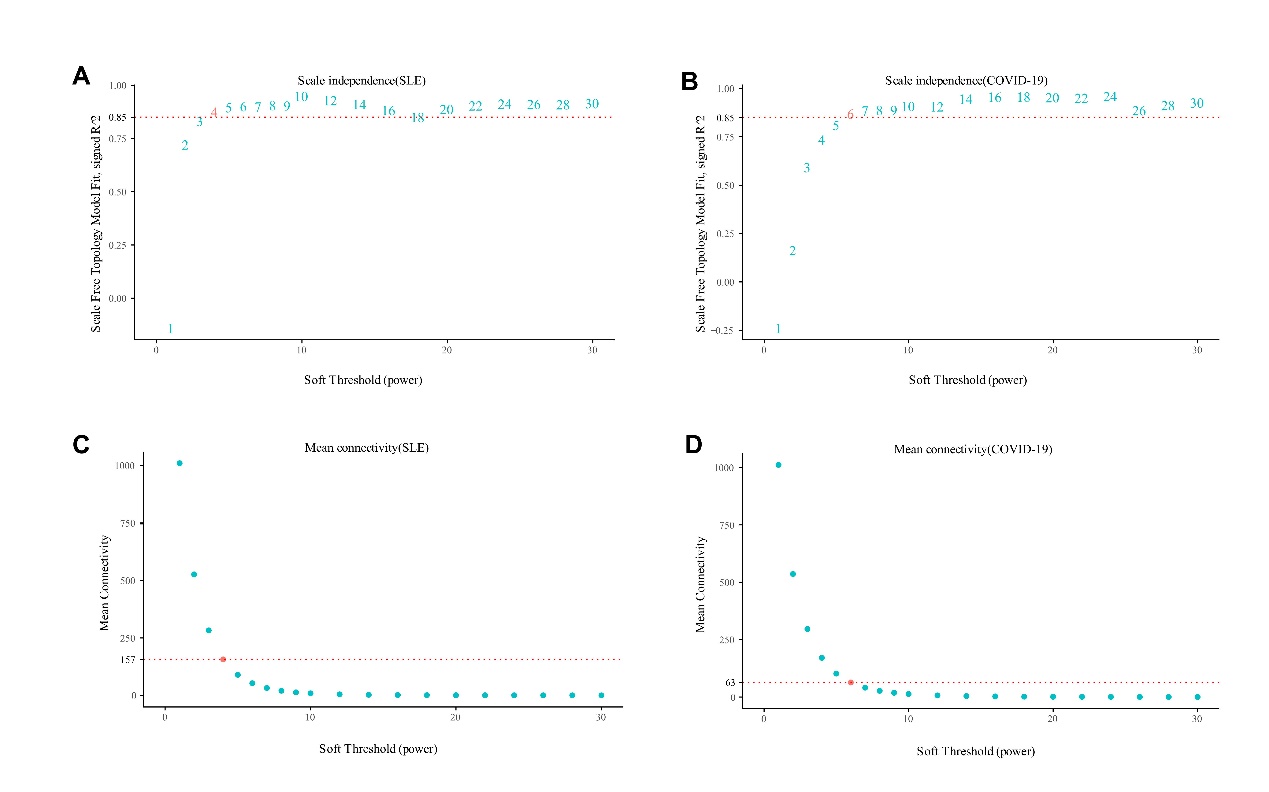
**

**Supplementary figure 2. Soft threshold power in WGCNA of bulk RNA-seq datasets from patients with SLE and COVID-19.** (A, B) Impact of soft threshold power on the scale-free topology fit index in WGCNA of patients with SLE and COVID-19; (C, D) Impact of soft threshold power on mean connectivity in WGCNA of patients with SLE and COVID-19.


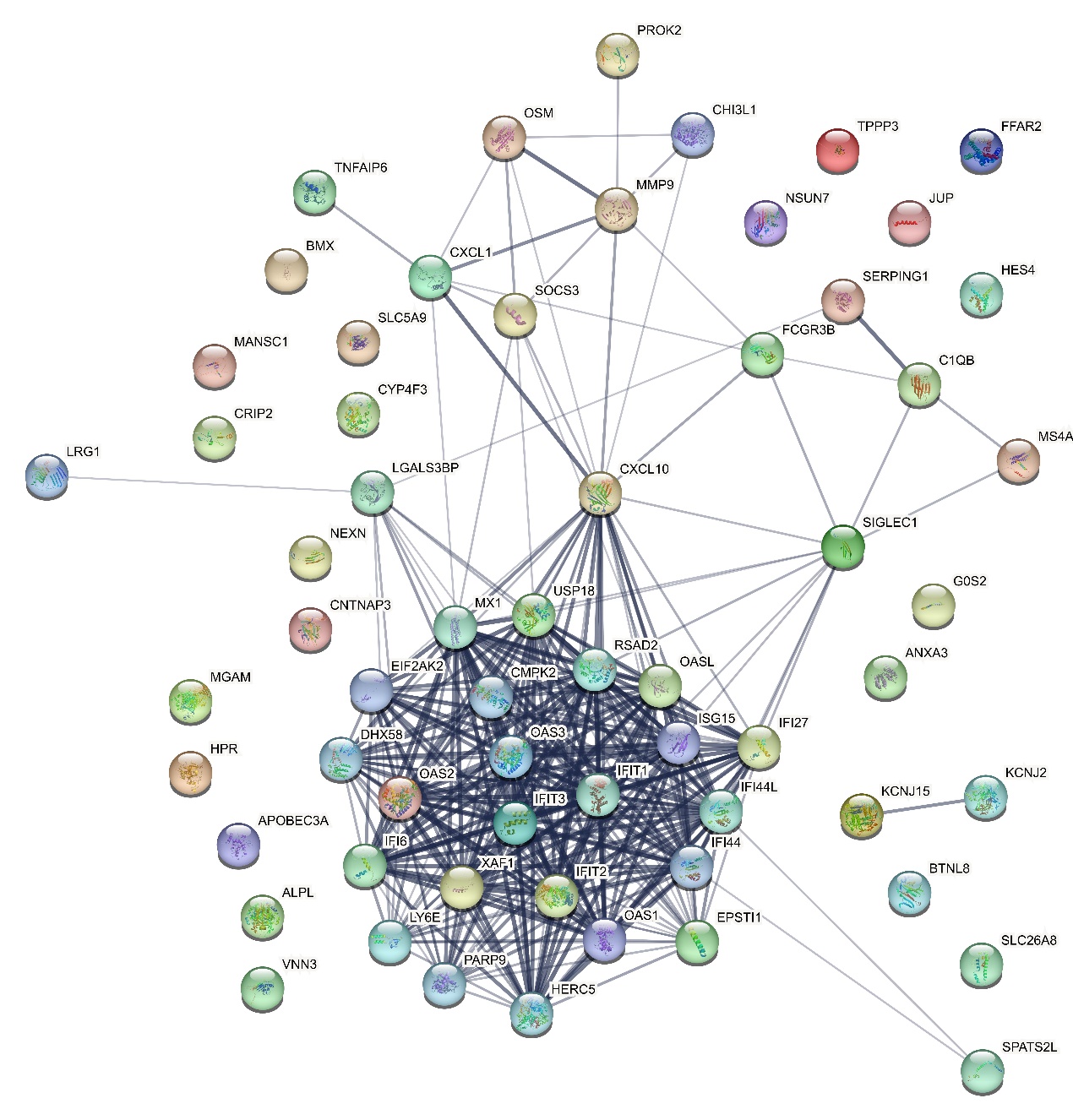


**Supplementary figure 3. PPI network of common genes with medium confidence.** The lines in the network represent interactions, and the thickness of the lines is directly proportional to the level of confidence.

**
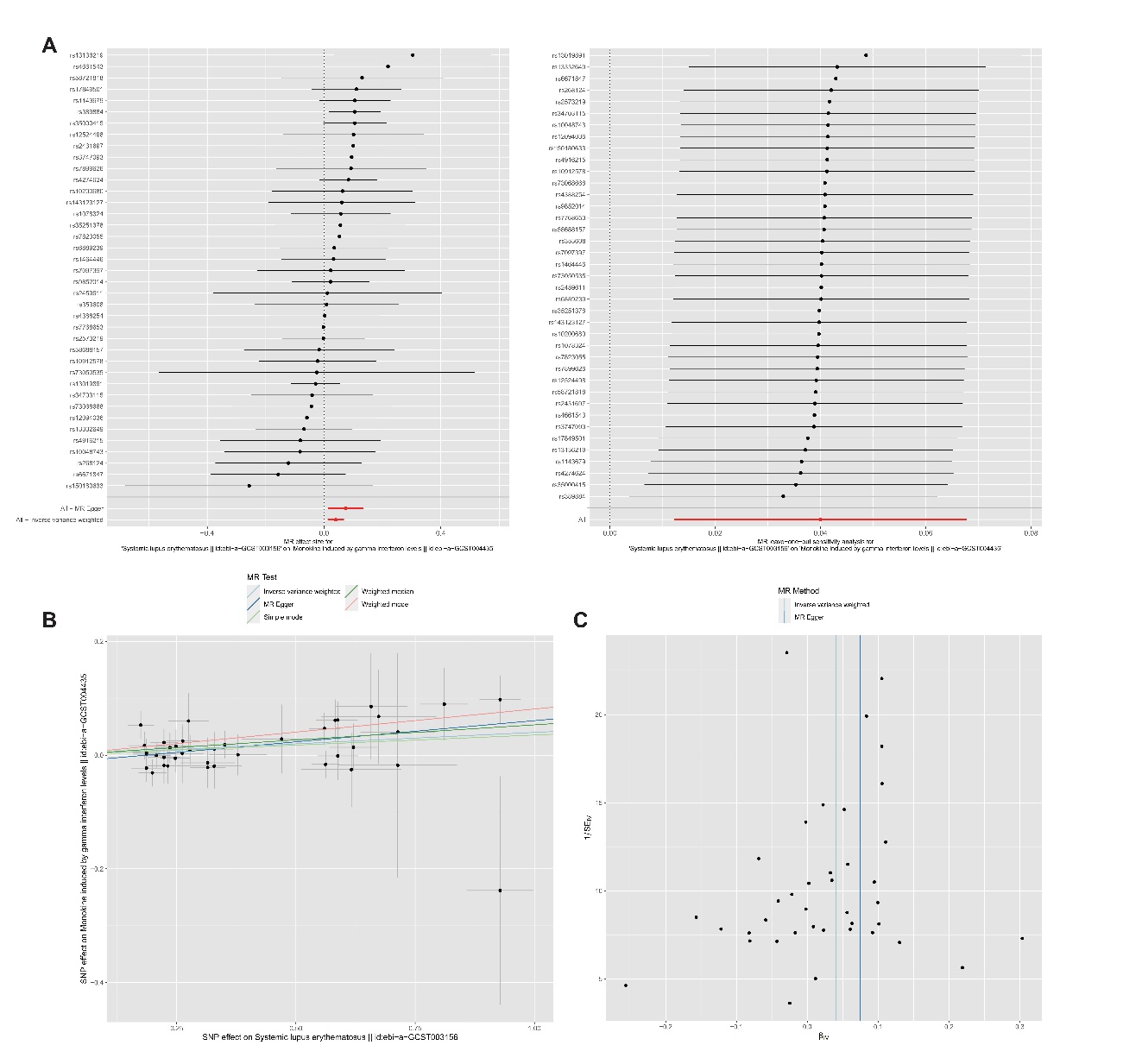
**

**Supplementary figure 4. Results of Leave-one-out, MR-Egger intercept, and Cochran’s Q analyses for MR sensitivity, pleiotropy, and heterogeneity testing.** (A) Forest plot shows the magnitude of causal effect and the results of leave-one-out sensitivity test after MR analysis. The horizontal axis and the corresponding horizontal line range of each point represent the magnitude of causal effect and the 95% confidence interval. (B) Scatter plot shows the causal effect results of each IV after five MR methods analysis. The two lines on each point represent the 95% confidence interval in the two directions of the IV on the horizontal and vertical axes. (C) Funnel plot shows the test results of publication bias in MR analysis. IV: instrumental variable.


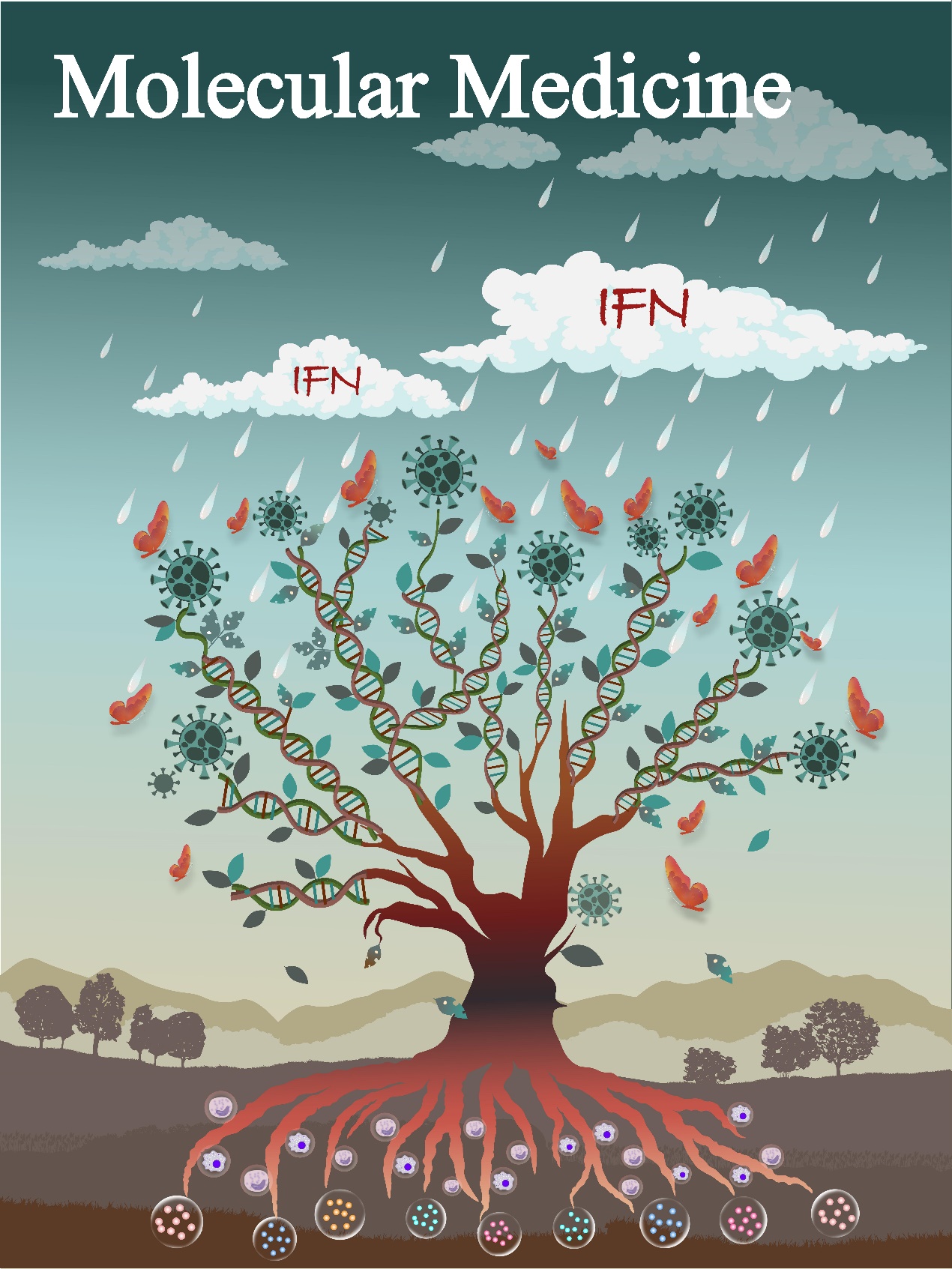


**Supplementary figure 5. Cover figure of the manuscript.** The butterflies refer to SLE.

| **Supplementary tables**  **Supplementary table 1. RNA-seq datasets from GEO.** | | | | | | |
| --- | --- | --- | --- | --- | --- | --- |
| **Index** | **GSE number** | **Platform** | **Samples** | **Source types** | **Disease name** | **Sequencing** |
| 1 | GSE50772 | GPL570 | 61 patients and 20 control | PBMC | SLE | Bulk |
| 2 | GSE122459 | GPL16791/ GPL18573 | 20 patients and 6 control | PBMC | SLE | Bulk |
| 3 | GSE179850 | GPL28038 | 31 patients and 16 control | PBMC | COVID-19 | Bulk |
| 4 | GSE121239 | GPL13158 | 292 patients and 20 control | PBMC | SLE | Bulk |
| 5 | GSE162577 | GPL24676 | 2 patients and 1 control | PBMC | SLE | Single-cell |
| 6 | GSE164805 | GPL26963 | 10 patients and 5 control | PBMC | COVID-19 | Bulk |
| 7 | GSE158055 | GPL24676/ GPL28038 | 171 patients and 25 control | PBMC | COVID-19 | Single-cell |
| 8 | GSE117928 | GPL14951 | 18 patients and 19 control | PBMC | SSc | Bulk |
| 9 | GSE25101 | GPL6947 | 16 patients and 16 control | Peripheral Blood | AS | Bulk |
| 10 | GSE3365 | GPL96 | 59 patients and 42 control | PBMC | CD | Bulk |
| 11 | GSE113469 | GPL10558 | 20 patients and 20 control | PBMC | Celiac | Bulk |
| 12 | GSE112943 | GPL10558 | 10 patients and 10 control | Skin | CLE | Bulk |
| 13 | GSE128470 | GPL96 | 12 patients and 12 control | Muscle | DM | Bulk |
| 14 | GSE112943 | GPL10558 | 14 patients and 7 control | Kidney | LN | Bulk |
| 15 | GSE159225 | GPL20795 | 30 patients and 20 control | Peripheral Blood | MS | Bulk |
| 16 | GSE21592 | GPL571 | 10 patients and 10 control | Peripheral Blood | Narcolepsy | Bulk |
| 17 | GSE128470 | GPL96 | 6 patients and 12 control | Muscle | NM | Bulk |
| 18 | GSE128470 | GPL96 | 7 patients and 12 control | Muscle | PM | Bulk |
| 19 | GSE192867 | GPL23126 | 62 patients and 10 control | PBMC | Psoriasis | Bulk |
| 20 | GSE90081 | GPL11154 | 12 patients and 12 control | Peripheral Blood | RA | Bulk |
| 21 | GSE40611 | GPL570 | 17 patients and 20 control | Parotid | SS | Bulk |
| 22 | GSE156035 | GPL20844 | 20 patients and 20 control | Peripheral Blood | Type 1 diabetes | Bulk |
| 23 | GSE3365 | GPL96 | 25 patients and 32 control | PBMC | UC | Bulk |
| 24 | GSE161664 | GPL21290 | 12 healthy subjects | SAEC | / | Bulk |
| 25 | GSE186460 | GPL16791 | 2 vitro organotypic models | Lung epithelial cells | COVID-19 | Bulk |
| 26 | GSE139940 | GPL21290 | 17 patients and 17 control | Peripheral Blood | SLE | Bulk |

| **Supplementary table 2. GWAS datasets from IEU OpenGWAS project.** | | | | | |
| --- | --- | --- | --- | --- | --- |
| **ID** | **Trait** | **Population** | **Sample** | **nControl** | **nSNP** |
| ebi-a-GCST010779 | COVID-19 (hospitalized vs population) RELEASE 4 | European | 908494 | 902088 | 11272365 |
| ebi-a-GCST010781 | COVID-19 (predicted covid from self-reported symptoms vs predicted or self-reported non-covid) RELEASE 4 | European | 38932 | 35728 | 11379674 |
| ebi-a-GCST011075 | COVID-19 (very severe respiratory confirmed vs population) RELEASE 5 | European | 1388342 | 1383241 | 9739225 |
| ebi-a-GCST010776 | COVID-19 (RELEASE 4) | European | 1299010 | 1284876 | 11435708 |
| ebi-a-GCST90000256 | Severe COVID-19 infection with respiratory failure (analysis II) | European | 3790 | 2180 | 8095992 |
| ebi-a-GCST011077 | COVID-19 (very severe respiratory confirmed vs population) RELEASE 5 | European | 1059456 | 1054664 | 7496658 |
| finn-b-SLE_NOS | Systemic lupus erythematosus, unspecified | European | 218696 | 218254 | 16380466 |
| ebi-a-GCST90014238 | Systemic lupus erythematosus | East Asian | 1506 | 994 | 6688607 |
| finn-b-SLE_OTH | Other forms of systemic lupus erythematosus | European | 218377 | 218254 | 16380466 |
| finn-b-DRUGADVERS_ SYSTEMIC_LUPUS_ERYTHEMAT | Drug-induced systemic lupus erythematosus | European | 218792 | 218691 | 16380466 |
| ebi-a-GCST003156 | Systemic lupus erythematosus | European | 14267 | 9066 | 7071163 |
| finn-b-L12_LUPUS | Lupus erythematosus | European | 207803 | 207482 | 16380444 |
| ebi-a-GCST004435 | Monokine induced by gamma interferon levels | European | 3685 | NA | 9579894 |
| ebi-a-GCST004456 | Interferon gamma levels | European | 7701 | NA | 9785363 |
| prot-a-1416 | Interferon-induced protein with tetratricopeptide repeats 2 | European | 3301 | NA | 10534735 |
| prot-a-1418 | Interferon alpha-10 | European | 3301 | NA | 10534735 |
| prot-a-1419 | Interferon alpha-10 | European | 3301 | NA | 10534735 |
| prot-a-1420 | Interferon alpha-14 | European | 3301 | NA | 10534735 |
| prot-a-1421 | Interferon alpha-4 | European | 3301 | NA | 10534735 |
| prot-a-1422 | Interferon alpha-5 | European | 3301 | NA | 10534735 |
| prot-a-1423 | Interferon alpha-6 | European | 3301 | NA | 10534735 |
| prot-a-1424 | Interferon alpha-7 | European | 3301 | NA | 10534735 |
| prot-a-1425 | Interferon alpha-8 | European | 3301 | NA | 10534735 |
| prot-a-1426 | Interferon alpha/beta receptor 1 | European | 3301 | NA | 10534735 |
| prot-a-1427 | Interferon beta | European | 3301 | NA | 10534735 |
| prot-a-1428 | Interferon gamma | European | 3301 | NA | 10534735 |
| prot-a-1429 | Interferon gamma | European | 3301 | NA | 10534735 |
| prot-a-1430 | Interferon gamma receptor 1 | European | 3301 | NA | 10534735 |
| prot-a-1431 | Interferon gamma receptor 2 | European | 3301 | NA | 10534735 |
| prot-a-1432 | Interferon gamma receptor 2 | European | 3301 | NA | 10534735 |
| prot-a-1433 | Interferon gamma receptor 2 | European | 3301 | NA | 10534735 |
| prot-a-1438 | Interferon omega-1 | European | 3301 | NA | 10534735 |
| prot-a-1599 | Janus kinase and microtubule-interacting protein 3 | European | 3301 | NA | 10534735 |
| prot-c-2989_17_2 | IFN-g | European | NA | NA | 501428 |
| prot-c-3497_13_2 | IFN-aA | European | NA | NA | 501428 |
| prot-c-3716_63_2 | IFN-g R1 | European | NA | NA | 501428 |
| prot-c-4396_54_1 | IFN-lambda 1 | European | NA | NA | 501428 |
| prot-c-4397_26_2 | IFN-lambda 2 | European | NA | NA | 501428 |
| ebi-a-GCST004440 | Interferon gamma-induced protein 10 levels | European | 3685 | NA | 9576881 |
| prot-a-1417 | Interferon-induced protein with tetratricopeptide repeats 3 | European | 3301 | NA | 10534735 |
| prot-a-1434 | Interferon lambda-1 | European | 3301 | NA | 10534735 |
| prot-a-1435 | Interferon lambda-1 | European | 3301 | NA | 10534735 |
| prot-a-1436 | Interferon lambda-2 | European | 3301 | NA | 10534735 |
| prot-a-1437 | Interferon lambda receptor 1 | European | 3301 | NA | 10534735 |
| prot-a-1566 | Interferon regulatory factor 1 | European | 3301 | NA | 10534735 |
| prot-a-1567 | Interferon regulatory factor 1 | European | 3301 | NA | 10534735 |
| prot-a-1568 | Interferon regulatory factor 2 | European | 3301 | NA | 10534735 |
| prot-a-1569 | Interferon regulatory factor 4 | European | 3301 | NA | 10534735 |
| prot-a-1570 | Interferon regulatory factor 6 | European | 3301 | NA | 10534735 |
| prot-a-1571 | Interferon regulatory factor 9 | European | 3301 | NA | 10534735 |
| prot-a-1598 | Tyrosine-protein kinase JAK2 | European | 3301 | NA | 10534735 |
| prot-a-2433 | Protein-tyrosine kinase 2-beta | European | 3301 | NA | 10534735 |

| **Supplementary table 3. Clinical information comparison between SLE and** **SARS-CoV-2-infected SLE outpatients** | | | | | | | |
| --- | --- | --- | --- | --- | --- | --- | --- |
| **Patients** | **SLE, N=50** | | **SLE+SARS-CoV-2, N=110** | | **P-value** | | |
| **Demographics** | | | | | | | |
| Age, y, mean (25%-75%) | 42 (36-54) | | 43 (32-51) | | 0.427 | | |
| Gender，female, N (%) | 49 (98.0) | | 101 (91.8) | | 0.123 | | |
| **Laboratory examination (median, 25%-75%)** | | | | | | | |
| Anti-dsDNA antibodies, N=13/63* | 9 (69.2) | | 34 (54.0) | | 0.370 | | |
| WBC, N=25/78* | 6.57 (4.85-6.57) | | 5.63 (4.41-7.67) | | 0.049* | | |
| PLC, N=32/84* | 188.5 (126.5-251.5) | | 221.5 (172-279) | | <0.001*** | | |
| C3, N=30/71* | 0.73 (0.62-0.96) | | 0.99 (0.80-1.13) | | 0.108 | | |
| C4, N=29/63* | 0.21 (0.07-0.34) | | 0.26 (0.19-0.33) | | 0.650 | | |
| LEU, N=29/63* | 16.5 (6-49) | | 5 (1-18) | | 0.019* | | |
| **Comorbidities, N (%)** | | | | | | | |
| Hypertension | 19 (38.0) | | 31 (28.2) | | 0.214 | | |
| Diabetes | 4 (8.0) | | 9 (8.2) | | 0.619 | | |
| PAH | 2 (4.0) | | 1 (0.9) | | 0.233 | | |
| Cardio-cerebrovascular disease | 6 (12.0) | | 14 (12.7) | | 0.897 | | |
| **Medication administration, N (%)** | | | | | | | |
| Hydroxychloroquine | 34 (68.0) | | 75 (68.2) | | 0.982 | | |
| Immunosuppressants | 36 (72.0) | | 58 (52.7) | | 0.022* | | |
| *N=a/b indicates that this examination came from a relieved SLE**–**COVID-19 comorbidity patient and b worsened comorbidity patients | | | | | | | |
|  | | | | | | |  |
| **Supplementary table 4. Clinical information comparison before and after contracting COVID-19 in hospitalized SLE patients (paired samples)** | | | | | | |  |
| **Patients** | | **SLE, N=28** | | **SLE+COVID-19, N=28** | | **P-value** |  |
| **SLEDAI-2K score (median, 25%-75%)** | | 3 (2-6) | | 2 (1-4) | | 0.859 |  |
| **Laboratory examination (median, 25%-75%)** | | | | | | |  |
| **Blood routine examination** | | | | | | |  |
| RBC | | 3.84 (3.03-4.35) | | 3.52 (3.05-4.20) | | 0.166 |  |
| HGB | | 117 (84-127) | | 105 (93-120) | | 0.207 |  |
| PLC | | 173 (103-214) | | 146 (118-186) | | 0.629 |  |
| WBC | | 5.81 (4.55-7.56) | | 4.19 (3.14-6.81) | | 0.368 |  |
| AEC | | 0.020 (0.010-0.100) | | 0.000 (0.000-0.010) | | 0.039* |  |
| ANC | | 3.79 (2.50-6.04) | | 3.19 (2.02-6.11) | | 0.694 |  |
| AMC | | 0.38 (0.29-0.59) | | 0.27 (0.20-0.53) | | 0.146 |  |
| ABC | | 0.010 (0.000-0.020) | | 0.000 (0.000-0.010) | | 0.045* |  |
| CRP | | 3.7 (1.9-67.9) | | 12.1 (3.0-50.9) | | 0.368 |  |
| ESR, N=13/13* | | 16 (3-23) | | 17 (12-29) | | 0.910 |  |
| **Biochemical examination of blood component, N=27/27*** | | | | | | |  |
| Total bilirubin, N=20/20* | | 10 (7-12) | | 8 (6-11) | | 0.148 |  |
| Albumin, N=23/23* | | 35.1 (30.4-38.7) | | 31.1 (26.9-36.6) | | 0.935 |  |
| ALT | | 16 (12-28) | | 17 (11-30) | | 0.129 |  |
| AST, N=26/26* | | 22 (17-26) | | 24 (19-36) | | 0.055 |  |
| ALP, N=22/22* | | 62 (46-83) | | 63 (44-93) | | 0.439 |  |
| GGT, N=22/22* | | 25 (17-54) | | 32 (22-65) | | 0.439 |  |
| Urea | | 9.3 (5.1-15.6) | | 9.4 (4.5-14.3) | | 0.773 |  |
| Creatinine | | 142 (63-419) | | 137 (69-577) | | 0.593 |  |
| Uric acid, N=23/23* | | 313 (234-494) | | 338 (266-427) | | 0.366 |  |
| Serum potassium, N=24/24* | | 4.11 (3.65-4.55) | | 3.80 (3.36-4.40) | | 0.970 |  |
| Serum sodium, N=24/24* | | 139 (136-141) | | 139 (135-141) | | 0.149 |  |
| Serum calcium, N=17/17* | | 2.17 (2.06-2.32) | | 2.22 (1.99-2.25) | | 0.638 |  |
| Serum phosphate, N=12/12* | | 1.41 (1.04-1.65) | | 0.99 (0.86-2.27) | | 0.112 |  |
| Total cholesterol, N=17/17* | | 4.88 (3.87-5.75) | | 4.47 (3.84-5.44) | | 0.995 |  |
| Triglycerides, N=17/17* | | 1.48 (0.84-2.36) | | 2.09 (0.99-3.30) | | 0.205 |  |
| HDL-C, N=17/17* | | 1.39 (1.11-1.79) | | 1.02 (0.96-1.35) | | 0.163 |  |
| LDL-C, N=17/17* | | 2.60 (2.01-3.14) | | 2.71 (2.21-3.66) | | 0.988 |  |
| Urinary protein, N=16/16* | | 0.5 (0-2) | | 2 (0-4) | | 0.102 |  |
| LEU, N=20/20* | | 6 (2-24) | | 7 (3-54) | | 0.647 |  |
| BNP, N=0/10* | | - | | 63 (28-345) | | / |  |
| NT-prBNP, N=3/3* | | 113.0 (41.9-127.5) | | 255.5 (131.5-411.0) | | 0.250 |  |
| **Immune-related, N=20/20*** | | | | | | |  |
| IgA | | 1.96 (1.34-3.06) | | 2.03 (1.64-2.38) | | 0.092 |  |
| IgG | | 10.84 (9.27-13.32) | | 9.49 (8.57-12.46) | | 0.083 |  |
| IgM | | 0.61 (0.49-1.32) | | 0.83 (0.45-1.66) | | 0.397 |  |
| C3, N=19/19* | | 0.75 (0.58-0.97) | | 0.79 (0.72-1.13) | | 0.832 |  |
| C4, N=16/16* | | 0.22 (0.09-0.25) | | 0.30 (0.18-0.43) | | 0.791 |  |
| Complement deficiency, N=16/16* | | 8 (42.1) | | 4 (21.1) | | 0.148 |  |
| Anti-dsDNA antibodies, N=9/9* | | 5 (55.6) | | 4 (44.4) | | 0.500 |  |
| **Cell composition, N=7/7*** | | | | | | |  |
| CD3+ T cells (%) | | 84.8 (78.3-89.3) | | 86.2 (73.5-88.5) | | 0.144 |  |
| CD4+ T cells (%) | | 35.9 (28.7-44.1) | | 27.7 (24.9-35.2) | | 0.059 |  |
| CD8+ T cells (%) | | 48.9 (38.3-52.6) | | 47.0 (41.5-63.6) | | 0.976 |  |
| CD4+ T cells/CD8+ T cells | | 0.7 (0.6-1.2) | | 0.6 (0.4-0.8) | | 0.113 |  |
| CD3-CD19+ B cells (%) | | 5.8 (1.8-7.2) | | 7.6 (0.4-8.4) | | 0.002** |  |
| CD3-CD56+ CD16+ NK cells (%) | | 11.4 (5.5-14.4) | | 12.4 (6.0-17.4) | | 0.259 |  |
| T lymphocyte, N=5/5* | | 475 (334-919) | | 638 (196-773) | | 0.029* |  |
| CD4+ T lymphocyte, N=5/5* | | 171 (130-491) | | 181 (85-259) | | <0.001*** |  |
| CD8+ T lymphocyte, N=5/5* | | 284 (195-432) | | 348 (110-553) | | 0.362 |  |
| B lymphocyte, N=5/5* | | 19 (10-73) | | 18 (3-53) | | 0.161 |  |
| NK cell, N=5/5* | | 47 (41-78) | | 45 (39-81) | | 0.001** |  |
| **Medication administration, N (%)** | | | | | | |  |
| Glucocorticoids | | 26 (100.0) | | 26 (100.0) | | / |  |
| Hydroxychloroquine | | 16 (61.5) | | 13 (50.0) | | 0.402 |  |
| Immunosuppressants | | 16 (61.5) | | 8 (30.8) | | 0.026* |  |
| Antiviral | | 2 (7.7) | | 19 (73.1) | | <0.001*** |  |
| Antibiotics | | 8 (30.8) | | 17 (65.4) | | 0.012* |  |
| Antipyretic analgesics | | 0 | | 6 (23.1) | | 0.011* |  |
| *: N=a/b indicates that this examination came from a relieved SLE**–**COVID-19 comorbidity patient and b worsened comorbidity patients | | | | | | |  |

| **Supplementary table 5. Clinical information comparison between SLE outpatients with mild and moderate/severe SARS-CoV-2 infection** | | | | | | |  |
| --- | --- | --- | --- | --- | --- | --- | --- |
| **SARS-CoV-2-infected SLE patients** | | **Mild, N=68** | | **Moderate/severe , N=42** | | **P-value** |  |
| **Demographics** | | | | | | |  |
| Age, y, mean (25%-75%) | | 44 (31-53） | | 41 (32-47） | | 0.165 |  |
| Gender, female, N (%) | | 62 (91.2) | | 39 (92.9) | | 0.528 |  |
| **Laboratory examination (median, 25%-75%)** | | | | | | |  |
| Anti-dsDNA antibodies, N=46/17* | | 21 (45.7) | | 13 (76.5) | | 0.045* |  |
| WBC, N=61/17* | | 5.36 (4.29-7.19) | | 6.35 (4.99-8.36) | | 0.118 |  |
| PLC, N=67/17* | | 207 (162-282) | | 235 (175-278) | | 0.429 |  |
| C3, N=55/16* | | 1.08 (0.84-1.14) | | 0.82 (0.68-1.10) | | 0.007** |  |
| C4, N=51/12* | | 0.29 (0.19-0.36) | | 0.20 (0.14-0.27) | | 0.180 |  |
| LEU, N=47/16* | | 5 (1-19) | | 5 (2-18) | | 0.456 |  |
| **Comorbidities, N (%)** | | | | | | |  |
| Hypertension | | 23 (33.8) | | 8 (19.0) | | 0.094 |  |
| Diabetes | | 5 (7.4） | | 4 (9.5) | | 0.472 |  |
| PAH | | 1 (1.5) | | 0 | | 0.615 |  |
| Cardio-cerebrovascular disease | | 9 (13.2） | | 5 (11.9) | | 0.839 |  |
| **Medication administration, N (%)** | | | | | | |  |
| Glucocorticoids | | 31 (45.6） | | 29 (69.0) | | 0.013** |  |
| Hydroxychloroquine | | 48 (70.6） | | 27 (64.3) | | 0.491 |  |
| Immunosuppressants | | 38 (55.9） | | 20 (47.6) | | 0.399 |  |
| *: N=a/b indicates that this examination came from a relieved SLE**–**COVID-19 comorbidity patient and b worsened comorbidity patients | | | | | | |  |
| **Supplementary table 6. GO analysis of hub genes from GeneMANIA.** | | | | | | | |
| **Index** | **Function** | | **FDR** | | **Genes in network** | | |
| 1 | response to type I interferon | | 5.93781E-41 | | 22 | | |
| 2 | cellular response to type I interferon | | 5.93781E-41 | | 22 | | |
| 3 | response to virus | | 1.81921E-30 | | 21 | | |
| 4 | regulation of viral genome replication | | 3.97193E-23 | | 15 | | |
| 5 | negative regulation of viral process | | 1.18386E-22 | | 15 | | |
| 6 | viral genome replication | | 1.82705E-22 | | 15 | | |
| 7 | regulation of viral life cycle | | 2.76251E-22 | | 16 | | |
| 8 | regulation of viral process | | 7.50808E-22 | | 17 | | |
| 9 | regulation of symbiotic process | | 1.62983E-21 | | 17 | | |
| 10 | viral life cycle | | 2.87468E-18 | | 16 | | |
| 11 | cellular response to interferon-gamma | | 2.23861E-07 | | 7 | | |
| 12 | response to interferon-gamma | | 5.6281E-06 | | 7 | | |
| 13 | regulation of nuclease activity | | 6.56388E-05 | | 4 | | |
| 14 | adenylyltransferase activity | | 0.000316553 | | 4 | | |
| 15 | regulation of type I interferon production | | 0.001322943 | | 5 | | |
| 16 | type I interferon production | | 0.00185766 | | 5 | | |
| 17 | negative regulation of viral life cycle | | 0.004362091 | | 3 | | |
| 18 | interferon-alpha production | | 0.010910387 | | 3 | | |
| 19 | regulation of interferon-alpha production | | 0.010910387 | | 3 | | |
| 20 | positive regulation of type I interferon production | | 0.024356308 | | 3 | | |
| 21 | nucleotidyltransferase activity | | 0.029296823 | | 4 | | |

| **Supplementary table 7. Top 10 drug signature of hub genes and core TFs according to combined score from CMAP.** | | | |
| --- | --- | --- | --- |
| **Drugs Name** | **P-value** | **Combined score** | **Genes** |
| Suloctidil | 3.25E-39 | 361,202.89 | RSAD2;STAT1;STAT2;MX1;IFI6;IFI44;ISG15;IFIT1;IFI44L;IFIT3;IFIT2; OAS1;OAS2;OAS3;IRF7;XAF1;IRF9 |
| Acetohexamide | 4.55E-27 | 36,063.31 | RSAD2;STAT1;MX1;IFI6;IFI44;IFIT1;IFI44L;IFIT3;OAS1;IFI27;OAS2;OAS3;IRF9 |
| Prochlorperazine | 4.55E-27 | 36,063.31 | STAT1;IFI6;IFI44;ISG15;IFIT1;IFIT3;OAS1;IFI27;OAS2;OAS3;IRF7;XAF1;IRF9 |
| Terfenadine | 2.23E-24 | 24,623.45 | OAS1;STAT1;MX1;IFI6;IRF7;IFI44;ISG15;IFIT1;XAF1;IFIT3;IRF9;IFIT2 |
| Prenylamine | 2.23E-24 | 24,623.45 | OAS1;STAT1;MX1;IFI6;IRF7;IFI44;ISG15;IFIT1;XAF1;IFIT3;IRF9;IFIT2 |
| Propofol | 8.55E-22 | 17,038.99 | OAS1;IFI27;STAT1;OAS3;IFI6;IRF7;ISG15;IFIT1;IFIT3;IRF9;IFIT2 |
| Econazole | 8.55E-22 | 17,038.99 | OAS1;STAT1;OAS2;MX1;IFI6;IFI44;IFIT1;IFI44L;IFIT3;IRF9;IFIT2 |
| Prasterone | 2.60E-19 | 11,822.80 | OAS1;STAT1;OAS2;OAS3;IFI6;ISG15;IFIT1;IFIT3;IRF9;IFIT2 |
| Niflumic acid | 2.60E-19 | 11,822.80 | OAS1;IFI27;STAT1;OAS2;OAS3;IFI6;IRF7;ISG15;IFIT1;IRF9 |
| Mephentermine | 6.32E-17 | 8,153.02 | OAS1;IFI27;STAT1;OAS2;OAS3;IFI6;ISG15;IFIT1;IRF9 |

| **Supplementary table 8. Results of MR analysis between SLE and COVID-19.** | | | | | | | | | | |
| --- | --- | --- | --- | --- | --- | --- | --- | --- | --- | --- |
| **Data Information** | | **COVID-19 as exposure** | | | | **SLE as exposure** | | | | |
| **Trait COVID-19** | **Trait SLE** | **nSNP** | **b** | **se** | **P-value** | **nSNP** | **b** | **se** | **P-value** |  |
| ebi-a-GCST010776 | ebi-a-GCST003156 | 2 | 0.015 | 0.156 | 0.923 | 41 | -0.018 | 0.009 | 0.051 |  |
| ebi-a-GCST010776 | ebi-a-GCST90014238 | 1 | -0.380 | 1.193 | 0.750 | 3 | -0.018 | 0.021 | 0.372 |  |
| ebi-a-GCST010776 | finn-b-DRUGADVERS_ SYSTEMIC_LUPUS_ERYTHEMAT | 5 | -1.164 | 0.618 | 0.126 | 0 | / | / | / |  |
| ebi-a-GCST010776 | finn-b-L12_LUPUS | 5 | 0.025 | 0.348 | 0.943 | 0 | / | / | / |  |
| ebi-a-GCST010776 | finn-b-SLE_NOS | 5 | -0.255 | 0.299 | 0.395 | 2 | -0.052 | 0.029 | 0.082 |  |
| ebi-a-GCST010776 | finn-b-SLE_OTH | 5 | -0.405 | 0.592 | 0.494 | 0 | / | / | / |  |
| ebi-a-GCST010779 | ebi-a-GCST003156 | 4 | -0.235 | 0.276 | 0.395 | 41 | -0.017 | 0.013 | 0.204 |  |
| ebi-a-GCST010779 | ebi-a-GCST90014238 | 5 | 0.227 | 0.308 | 0.461 | 3 | 0.001 | 0.037 | 0.970 |  |
| ebi-a-GCST010779 | finn-b-DRUGADVERS_ SYSTEMIC_LUPUS_ERYTHEMAT | 7 | -0.460 | 0.310 | 0.138 | 0 | / | / | / |  |
| ebi-a-GCST010779 | finn-b-L12_LUPUS | 7 | 0.023 | 0.240 | 0.924 | 0 | / | / | / |  |
| ebi-a-GCST010779 | finn-b-SLE_NOS | 7 | -0.042 | 0.166 | 0.800 | 2 | -0.058 | 0.036 | 0.110 |  |
| ebi-a-GCST010779 | finn-b-SLE_OTH | 7 | -0.246 | 0.277 | 0.374 | 0 | / | / | / |  |
| ebi-a-GCST010781 | ebi-a-GCST003156 | 0 | / | / | / | 41 | -0.006 | 0.019 | 0.770 |  |
| ebi-a-GCST010781 | ebi-a-GCST90014238 | 0 | / | / | / | 3 | -0.032 | 0.048 | 0.511 |  |
| ebi-a-GCST010781 | finn-b-DRUGADVERS_ SYSTEMIC_LUPUS_ERYTHEMAT | 0 | / | / | / | 0 | / | / | / |  |
| ebi-a-GCST010781 | finn-b-L12_LUPUS | 0 | / | / | / | 0 | / | / | / |  |
| ebi-a-GCST010781 | finn-b-SLE_NOS | 0 | / | / | / | 2 | 0.008 | 0.048 | 0.871 |  |
| ebi-a-GCST010781 | finn-b-SLE_OTH | 0 | / | / | / | 0 | / | / | / |  |
| ebi-a-GCST011075 | ebi-a-GCST003156 | 6 | -0.122 | 0.138 | 0.378 | 41 | -0.047 | 0.019 | 0.434 |  |
| ebi-a-GCST011075 | ebi-a-GCST90014238 | 6 | 0.170 | 0.202 | 0.398 | 3 | 0.006 | 0.069 | 0.934 |  |
| ebi-a-GCST011075 | finn-b-DRUGADVERS_ SYSTEMIC_LUPUS_ERYTHEMAT | 8 | -0.298 | 0.286 | 0.296 | 0 | / | / | / |  |
| ebi-a-GCST011075 | finn-b-L12_LUPUS | 8 | 0.112 | 0.143 | 0.433 | 0 | / | / | / |  |
| ebi-a-GCST011075 | finn-b-SLE_NOS | 8 | -0.059 | 0.168 | 0.724 | 2 | -0.078 | 0.043 | 0.067 |  |
| ebi-a-GCST011075 | finn-b-SLE_OTH | 8 | -0.199 | 0.218 | 0.359 | 0 |  |  |  |  |
| ebi-a-GCST011077 | ebi-a-GCST003156 | 6 | -0.114 | 0.142 | 0.420 | 41 | -0.037 | 0.018 | 0.088 |  |
| ebi-a-GCST011077 | ebi-a-GCST90014238 | 5 | 0.418 | 0.289 | 0.149 | 2 | 0.027 | 0.113 | 0.813 |  |
| ebi-a-GCST011077 | finn-b-DRUGADVERS_ SYSTEMIC_LUPUS_ERYTHEMAT | 7 | -0.302 | 0.316 | 0.338 | 0 | / | / | / |  |
| ebi-a-GCST011077 | finn-b-L12_LUPUS | 7 | 0.147 | 0.144 | 0.308 | 0 | / | / | / |  |
| ebi-a-GCST011077 | finn-b-SLE_NOS | 7 | 0.010 | 0.145 | 0.946 | 1 | -0.003 | 0.021 | 0.879 |  |
| ebi-a-GCST011077 | finn-b-SLE_OTH | 7 | -0.166 | 0.227 | 0.463 | 0 |  |  |  |  |
| ebi-a-GCST90000256 | ebi-a-GCST003156 | 0 | / | / | / | 39 | -0.023 | 0.041 | 0.572 |  |
| ebi-a-GCST90000256 | ebi-a-GCST90014238 | 0 | / | / | / | 3 | -0.043 | 0.153 | 0.777 |  |
| ebi-a-GCST90000256 | finn-b-DRUGADVERS_ SYSTEMIC_LUPUS_ERYTHEMAT | 1 | -0.566 | 0.347 | 0.103 | 0 | / | / | / |  |
| ebi-a-GCST90000256 | finn-b-L12_LUPUS | 1 | 0.064 | 0.195 | 0.741 | 0 | / | / | / |  |
| ebi-a-GCST90000256 | finn-b-SLE_NOS | 1 | -0.144 | 0.169 | 0.393 | 2 | 0.061 | 0.080 | 0.444 |  |
| ebi-a-GCST90000256 | finn-b-SLE_OTH | 1 | -0.353 | 0.310 | 0.255 | 0 | / | / | / |  |

| **Supplementary table 9. Clinical information comparison between hospitalized SLE–COVID-19 patients diagnosed as CRS and non-CRS** | | | |
| --- | --- | --- | --- |
| **SLE–COVID-19 comorbidity patients** | **Non-CRS, N=7** | **CRS, N=27** | **P-value** |
| **Demographics** | | | |
| Age, y, mean (25%–75%) | 50 (35-55) | 56 (44-58) | 0.191 |
| Gender, female, N (%) | 6 (85.7) | 24 (88.9) | 0.622 |
| **Clinical manifestations of COVID-19, N (%)** | | | |
| Fever | 5 (71.4) | 21 (77.8) | 0.535 |
| Cough and expectoration | 5 (71.4) | 22 (81.5) | 0.450 |
| Lassitude | 3 (42.9) | 7 (25.9) | 0.330 |
| Muscle soreness | 3 (42.9) | 3 (11.1) | 0.086 |
| Runny and stuffy nose | 2 (28.6) | 1 (3.7) | 0.101 |
| Pharyngalgia and itchy throat | 1 (14.3) | 3 (11.1) | 0.622 |
| Abdominal pain and diarrhea | 0 | 2 (7.4) | 0.626 |
| Nausea and vomiting | 0 | 4 (14.8) | 0.378 |
| Tachypnea and chest distress | 2 (28.6) | 9 (33.3) | 0.596 |
| Dizziness and headache | 0 | 1 (3.7) | 0.794 |
| **SLEDAI-2K score (median, 25%-75%)** | 1 (1-7) | 2 (1-4) | 0.868 |
| **Comorbidities, N (%)** | | | |
| Hypertension | 4 (57.1) | 18 (66.7) | 0.479 |
| Diabetes | 0 | 5 (18.5) | 0.290 |
| PAH | 0 | 4 (14.8) | 0.378 |
| Cardio-cerebrovascular disease | 1 (14.3) | 6 (22.2) | 0.550 |
| **Laboratory examination (median, 25%-75%)** | | | |
| **Blood routine examination** | | | |
| RBC | 4.03 (2.72-4.68) | 3.43 (3.00-3.71) | 0.151 |
| HGB | 120 (78-123) | 104 (90-114) | 0.294 |
| PLC | 178 (71-232) | 142 (118-194) | 0.617 |
| WBC | 8.18 (5.21-9.71) | 4.02 (3.10-6.00) | 0.030* |
| AEC | 0.000 (0.000-0.080) | 0.000 (0.000-0.010) | 0.427 |
| ANC | 4.93 (4.34-7.76) | 3.01 (1.97-5.33) | 0.139 |
| AMC | 0.46 (0.35-0.69) | 0.26 (0.18-0.48) | 0.048* |
| ABC | 0.010 (0.000-0.030) | 0.000 (0.000-0.010) | 0.172 |
| CRP, N=5/25* | 12.1 (3.7-23.3) | 28.3 (18.1-91.1) | 0.037* |
| ESR, N=6/18* | 21.5 (9.3-36.8) | 18.5 (10.8-30.0) | 0.673 |
| **Biochemical examination of blood component** | | | |
| Total bilirubin, N=7/24* | 7 (6-13) | 8 (7-11) | 0.811 |
| Albumin, N=7/24* | 38.6 (32.6-41.7) | 30.1 (26.9-34.6) | 0.038* |
| ALT | 17 (15-36) | 20 (14-32) | 0.677 |
| AST, N=7/26* | 24 (16-30) | 25 (20-49) | 0.620 |
| ALP, N=7/24* | 88 (55-109) | 63 (42-89) | 0.076 |
| GGT, N=7/24* | 26 (23-35) | 32 (19-86) | 0.661 |
| Urea | 5.4 (4.3-18.1) | 9.8 (4.2-22.3) | 0.097 |
| Creatinine | 69 (55-753) | 97 (66-355) | 0.163 |
| Uric acid, N=6/22* | 277 (255-350) | 373 (252-453) | 0.530 |
| Serum potassium, N=7/26* | 3.90 (3.63-4.16) | 4.02 (3.31-4.39) | 0.880 |
| Serum sodium, N==7/26* | 139 (138-142) | 136 (134-142) | 0.232 |
| Serum calcium, N=6/22* | 2.23 (2.08-2.45) | 2.13 (1.96-2.22) | 0.059 |
| Serum phosphate, N=5/14* | 1.11 (0.72-1.80) | 1.07 (0.91-1.64) | 0.754 |
| Total cholesterol, N=6/19* | 4.66 (4.20-5.22) | 4.07 (3.51-5.01) | 0.877 |
| Triglycerides, N=6/19* | 2.32 (1.15-3.89) | 1.80 (1.05-2.31) | 0.366 |
| HDL-C, N=6/19* | 1.12 (0.97-1.49) | 1.00 (0.94-1.09) | 0.246 |
| LDL-C, N=6/19* | 2.74 (2.30-3.42) | 2.35 (1.92-3.27) | 0.926 |
| Urinary protein, N=6/22* | 2 (33.3) | 7 (31.8) | 0.650 |
| LEU, N=5/22* | 9 (7-12) | 4 (1-59) | 0.377 |
| BNP, N=3/7* | 23 (17-26) | 126 (47-969) | 0.017* |
| NT-prBNP, N=4/17* | 89.6 (57.5-116.0) | 909.0 (203.0-7513.5) | 0.006** |
| **Immune-related, N=7/20*** | | | |
| IgA, N=6/19* | 2.02 (1.78-3.55) | 1.98 (1.42-2.22) | 0.092 |
| IgG, N=6/19* | 10.10 (7.38-14.54) | 9.28 (7.82-12.05) | 0.333 |
| IgM, N=6/19* | 0.50 (0.43-1.13) | 0.78 (0.43-1.41) | 0.534 |
| C3, N=6/19* | 0.99 (0.52-1.31) | 0.87 (0.74-1.16) | 0.514 |
| C4, N=6/19* | 0.29 (0.12-0.31) | 0.29 (0.19-0.43) | 0.721 |
| Complement deficiency, N=6/19* | 1 (16.7) | 5 (26.3) | 0.547 |
| Anti-dsDNA antibodies, N=6/18* | 3 (50.0) | 4 (22.2) | 0.215 |
| **Cell composition, N=6/18*** | | | |
| CD3+ T cells (%), N=5/17* | 74.1 (63.0-85.1) | 75.3 (57.1-86.2) | 0.704 |
| CD4+ T cells (%), N=5/17* | 30.2 (22.8-37.4) | 29.7 (23.6-35.6) | 0.595 |
| CD8+ T cells (%), N=5/17* | 44.4 (27.6-60.9) | 44.5 (31.9-55.9) | 0.969 |
| CD4+ T cells/CD8+ T cells, N=5/17* | 0.79 (0.37-1.36) | 0.71 (0.63-0.91) | 0.762 |
| CD3-CD19+ B cells (%), N=5/17* | 12.5 (7.9-16.5) | 7.6 (1.9-18.2) | 0.493 |
| CD3-CD56+ CD16+ NK cells (%), N=5/17* | 13.0 (5.7-20.2) | 12.4 (6.0-22.4) | 0.762 |
| T lymphocyte, N=4/15* | 400 (76-766) | 228 (194-497) | 0.920 |
| CD4+ T lymphocyte, N=4/15* | 130 (49-236) | 136 (63-184) | 0.960 |
| CD8+ T lymphocyte, N=4/15* | 281 (40-535) | 125 (101-271) | 1.000 |
| B lymphocyte, N=4/15* | 61 (52-77) | 18 (5-76) | 0.152 |
| NK cell, N=4/15* | 50 (16-87) | 53 (27-74) | 0.961 |
| **Medication administration, N (%)*** | | | |
| Glucocorticoids | 7 (100.0) | 27 (100.0) | / |
| Hydroxychloroquine | 3 (42.9) | 14 (51.9) | 0.500 |
| Immunosuppressants | 3 (42.9) | 8 (29.6) | 0.404 |
| Antiviral | 4 (57.1) | 19 (70.4) | 0.404 |
| Antibiotics | 3 (42.9) | 20 (74.1) | 0.132 |
| Antipyretic analgesics | 0 | 6 (22.2) | 0.220 |
| Traditional Chinese medicine preparations | 2 (28.6) | 7 (25.9) | 0.614 |
| *: N=a/b indicates that this examination came from a relieved SLE**–**COVID-19 comorbidity patients and b worsened comorbidity patients | | | |
